# Supplementary material for: Macrophage-Induced Blood Vessels Guide Schwann Cell-Mediated Regeneration of Peripheral Nerves
Source: Cell. 2015 Aug 27;162(5):1127–39. doi: 10.1016/j.cell.2015.07.021 (PMC4553238; doi:10.1016/j.cell.2015.07.021)
Supplement: Document S1. Supplemental Experimental Procedures [file mmc1.pdf]

Cell

Supplemental Information

Macrophage-Induced Blood Vessels Guide Schwann Cell-Mediated Regeneration of Peripheral Nerves

Anne-Laure Cattin, Jemima J. Burden, Lucie Van Emmenis, Francesca E. Mackenzie, Julian J.A. Hoving, Noelia Garcia Calavia, Yanping Guo, Maeve McLaughlin, Laura H. Rosenberg, Victor Quereda, Denisa Jamecna, Ilaria Napoli, Simona Parrinello, Tariq Enver, Christiana Ruhrberg, and Alison C. Lloyd

Supplemental Experimental Procedures

In vivo analysis

Nerve injury

To induce the nerve transection injuries, left (rats) and right (mice) sciatic nerves were exposed under general anesthesia in aseptic conditions and transected with scissors at mid-thigh. Nerves were dissected at indicated time-points following transection for analysis. Cell proliferation was determined by measuring 5-ethynyl-2'-deoxyuridine (EdU) (Invitrogen) incorporation following a single intra-peritoneal injection of EdU (25mg/Kg body weight), 12 hours before sacrificing the animals.

In vivo inhibition of VEGFR

To inhibit VEGFR prior to vascularization, PLP-EGFP transgenic mice were orally administrated 3 doses of Cabozantinib (100mg/kg) (Bentzien et al., 2013; Yakes et al., 2011) or vehicle every 12 hours, Day 4 following sciatic nerve transection (1st dose in the morning) and sciatic nerves were collected the next day (Day 5, evening) to analyse blood vessel density in the bridge. To inhibit VEGFR post-vascularisation, PLP-EGFP mice were administrated the same doses, Day 5 following injury (1st dose in the morning) and sciatic nerves were collected the next day (Day 6, evening) to analyse Schwann cell migration and axonal regrowth. To test whether inhibition of bridge vascularisation has an effect on Schwann cell migration and axonal regrowth, PLP-EGFP mice were orally administrated 4 doses of Cabozantinib or vehicle every 12 hours, Day 4 following sciatic nerve transection (1st dose in the morning) and sciatic nerves were dissected on Day 7.

Rescue experiment

Day 4 after sciatic nerve transection of *Vegfa$^{fl/fl}$ Tie2-Cre* mutant mice, nerves were re-exposed under general anesthesia in aseptic conditions and each bridge was injected with 5μl of VEGF-A^{188} (ReliaTech GmbH) (4μg/ml) or PBS using a hand-held glass capillary coupled to a Hamilton syringe mounted to a microinjector (Sutter Instrument). One day later, sciatic nerves were collected for analysis.

VEGF-A treatment to misdirect blood vessels

Prior to surgery, agarose beads coated with heparin (Sigma) were pre-incubated with either PBS or human recombinant VEGF-A^{165} (1μg/ml) for 4 hours. The left sciatic nerves of rats were exposed under general anesthesia in aseptic conditions and transected at mid-thigh. VEGF or PBS beads were then placed to the side of the injury site within adjacent muscle. 6 days later, sciatic nerves were collected for analysis.

Bone marrow transplantation

3×10^6 total bone marrow cells from donor mice were injected into the tail vein of lethally irradiated (10 Gy) B6SJCD45.1 recipient mice. Donor cells were harvested from bone marrow from 3 *Vegfa$^{fl/fl}$ Tie2-Cre* mutant mice and 3 *Vegfa$^{fl/fl}$* littermates, which also constitutively expressed YFP in all cells. The donor cells were analysed by FACS for the expression of the CD45.2 locus and YFP reporter before transplantation into WT mice, which all expressed the CD45.1 locus to enable the analysis of the efficiency of the transplant. Bone marrow cells of each mouse were injected into the tail vein of 3 recipient mice. In order to check the engraftment efficiency, peripheral blood was collected from each recipient after 4 weeks of transplantation and analysed by FACS for CD45 locus and YFP expression. FACS analysis of peripheral blood and bone marrow was performed as previously described (Karlsson et al., 2013). Briefly, red blood cells were lysed using ACK buffer. Nucleated cells were stained with CD45.1 and CD45.2 antibodies conjugated with APC, PE, PEcy7 or APC-AF700. Hoechst 33358 was used to exclude dead cells. Stained cells were analysed on a Gallios (Beckman Coulter) flow cytometer.

Mice with higher than 90% engraftment of donor cells, as determined by expression of the CD45.2 locus, were used for sciatic nerve transection. The efficiency of the transplant was further confirmed by YFP-expression of bone marrow cells of transplanted animals and the high percentage of YFP+ macrophages in the bridge following injury.

In vitro analysis

Ex vivo bridge analysis

Sensitivity to hypoxia

Rat nerve bridges at Day 2 following injury were collected and digested with 1mg/ml dispase and 1mg/ml collagenase for 45 minutes at 37ºC, washed 3 times in RPMI (Gibco) containing 10% FCS and plated on PLL and fibronectin coated coverslips at 6 $\times10^4$ cells/well in RPMI plus 10% FCS. After centrifugation at 300g for 5mins, cells were incubated overnight at 20% O_2, 5% CO_2. 100μM pimonidazole HCl (hypoxyprobe-1) was then added and the cells incubated at 20%, 1.5% or 0.1% O_2 for 4 hours before washing once with RPMI plus serum. Cells were then fixed in 4% paraformaldehyde (PFA) in PBS and immunostained for hypoxyprobe-1, Iba1 and the nuclei counterstained with Hoechst. For each experiment, each condition was in duplicate and 300 to 500 cells were counted per condition for each experiment.

Transwell migration assay

Cells from rat nerve bridges at Day 2 were purified as described above and seeded at 1×10^5 cells/well into the bottom of transwells overnight. The cells were then washed twice in

minimal media (RPMI containing BSA 100μg/ml, progesterone 60ng/ml, putrescine 16μg/ml, selenium 60ng/ml, thyroxine 50ng/ml, Triiodothyronine 50ng/ml, transferrin 100μg/ml, insulin 100ng/ml) and incubated at 1.5% O_2 for 24 hours. Minimal media was used as a control and 5ng/ml VEGF-A^{165} (Lonza) and 10% FCS were added as positive controls. Hanging cell culture inserts (8μm pore size, Millipore) were coated with fibronectin before adding Schwann cells or HUVECs ($7x10^4$ or $5x10^4$ cells respectively) in the presence or absence of 10μM Cabozantinib (Selleckchem) at 20% O_2. 4 hours later, cells on the upper surface were removed mechanically and cells that had migrated onto the lower surface were fixed in 4% PFA/PBS, the nuclei counterstained with Hoechst were then counted. 10 fields per condition were quantified, 5 from each duplicate and were normalised against migration in minimal media.

siRNA knockdown

siRNA knockdown was performed in primary rat Schwann cells using HiPerFect (Qiagen). siRNA target sequences used at the indicated concentrations were:
beta1 integrin siRNA 1 5'-ACAGCTGATTATCGATGCCTA-3' at 5nM
beta1 integrin siRNA 2 5'-CAAATTGTGGGTGGTGTACAA-3' at 5nM
Talin 1 siRNA 1 5'-CTGAGCGTCGTACAGAATCTA-3' at 3nM
Talin 1 siRNA 2 5'-CTGGTCGCTTGCAAGGTCAAA-3' at 3nM
Talin 2 siRNA 1 5'-ACCGGGCAAGCTTCTGACTAT-3' at 5nM
Talin 2 siRNA 2 5'-ACCCGAGGAATCAATAAGAAT-3' at 5nM
Following knockdown with beta1 integrin siRNA, cells were lysed in RIPA buffer (1% triton, 0.5% Na deoxycholate, 1mM EGTA, 50mM Tris pH 7.5) and standard Western Blotting for beta1 integrin was performed to validate the efficiency of the oligos at 36 hours. Following knockdown with talin 1 and talin 2 siRNA, cells were fixed and immunostained for talin to validate the efficiency of the oligos at 60 hours.

qPCR analysis

Nerve stump or nerve bridge samples were collected Day 2 or Day 5 following injury in rats and mice respectively and frozen in liquid nitrogen. Samples were crushed and homogenised on dry ice and then lysed in Trizol Reagent (Ambion). After RNA purification, RNA was reverse-transcribed using Super-Script II Reverse Transcriptase (Invitrogen). Quantitative PCR was then performed using the MESA Blue qPCR Kit (Eurogentec). Relative expression values for each gene of interest were obtained by normalizing to B2M (Napoli et al., 2012).
Primers sequence used for qPCR were:

Rat Vegfa Fwd: 5’-gagttaaacgaacgtacttgcaga-3’; Rev: 5’-tctagttcccgaaaccctga-3’
Mouse Vegfa Fwd: 5’-agaaggagagcagaagtccca-3’; Rev: 5’-gtccaccagggtctcaatcg-3’

In situ hybridisation

12μm cryosections of contralateral or transected rat sciatic nerves at Day 2 were fixed for 20 minutes in 4% PFA/PBS and permeabilised with 0.3% Triton-X in PBS for 20 minutes at room temperature. Sections were then acetylated for 10 minutes and pre-hybridised in the hybridisation solution (Sigma) for 2 hours at room temperature. Hybridisation was performed using the scramble-ISH, the positive probe and the VEGF-A LNA-probe (5DigN/AGGGAAGTAGATCACAGAGGTT/3DigN) (Exiqon) at 14nM overnight at 55°C. After hybridisation, the sections were washed with 5X SSC for 5 minutes, 2X SSC for 1 minute, 0.2X SSC + 50% formamide for 30 minutes at 55°C and finally 0.2X SSC for 5 minutes at room temperature, incubated for 1 hour at room temperature in 1% blocking solution (Roche) prior to incubation with the CD68 antibody overnight at 4°C. Slides were then incubated with anti-DIG-AP, 488 conjugated anti-rat secondary antibody and Hoechst for 1 hour at room temperature. To detect the probe, sections were incubated with Fast Red (Roche) for 2 hours at room temperature. Images were detected using confocal microscopy.

Schwann cell migration assays

2D migration assay

$8x10^3$ siRNA treated GFP-labelled Schwann cells were seeded into laminin-coated wells and incubated overnight before starting time-lapse phase microscopy.

Fibrin gel bead assay

The fibrin gel bead assay was performed as described in the protocol of Nakatsu et al., 2003, with some modifications. Specifically, after coating the beads with HUVECs, the beads were resuspended in 2.5 mg/ml fibrinogen containing aprotinin at 0.15 U/ml. GFP-expressing rat Schwann cells were then added to the beads at a final concentration of $1.25x10^4$ cells/ml. 0.625 U/mL of thrombin was added to promote fibrin formation and then 400μl of the solution was added to a 10mm glass-bottom dish or a 24-well plate (MatTek Corporation). Time-lapse microscopy or immunofluorescence analysis was performed 10 days later. Rho kinase inhibitor (Y-27632, Abcam) was used at 50μM, myosin-II inhibitor blebbistatin (Abcam) at 2μM and latrunculin B (Sigma) at 0.2μM.

Matrigel assay

Briefly, 150μl matrigel/well (BD Bioscience) was coated on a 24 well plate on ice and polymerised at 37°C for 30 min. $2.5x10^4$ HUVECS were seeded onto the matrigel and incubated for 14 hours to allow tubules to form. $5x10^3$ Scrambled or siRNA treated GFP-

labelled Schwann cells were seeded onto the tubules and imaged using time-lapse microscopy.

Time-lapse microscopy and analysis

Live-imaging was performed using a Zeiss Axiovert 200M microscope equipped with a Hamamatsu Orca AG camera controlled by Volocity software (Improvision) and an environmental chamber which maintained the temperature at 37°C and provided a humidified stream of 5% or 10% CO_2 in air. Images were taken every 10 minutes for 12 to 24 hours for 2D and fibrin gel experiments. To compare single cell behaviour on 2D laminin-coated surfaces with 3D fibrin gels, cells were tracked using Volocity software for 8 to 12 hours and then speed and directional persistence were measured as previously described (Gorelik and Gautreau, 2014). In matrigel experiments, images were acquired every 10 minutes for 24 hours, cells were tracked the first 6 to 8 hours of acquisition and the velocity was measured using Volocity software. GFP-positive Schwann cells were seeded 36 hours and 60 hours following knockdown of beta1 integrin and talins respectively.

When fibrin gels were treated with inhibitors, images were taken every 7.5 minutes 6 hours prior to and 6 hours after addition of inhibitors. The rear of the migrating cells was tracked and the velocity was measured using Volocity software (27 cells were measured with Y-27632, 35 cells with blebbistatin and 15 cells with latrunculin B from 2 separate experiments). For protrusion measurements, the length of 2 to 5 protrusions were measured and averaged per cell at 0 and 3 hours in the presence of each inhibitor. The velocity of the protrusions was then calculated for each cell (10 cells from 2 separate experiments were measured in each condition).

Immunostaining

Sciatic nerves were dissected and fixed for 4 hours in 4% PFA/PBS, cryoprotected in 30% sucrose/PBS overnight at 4°C, incubated in 50% OCT/30% sucrose/PBS for 2 hours and finally embedded in OCT before being frozen in liquid nitrogen.

Longitudinal cryosections of contralateral or cut sciatic nerves at indicated times after transection were analysed in both rats and mice. Sections for immunostaining (12μm) or for quantification of blood vessel density, Schwann cell migration and axonal regrowth (40-60μm) were cut using a cryostat (Leica). Thin sections (12μm) were permeabilised in 0.3% triton/PBS for 30 minutes, washed and then blocked in 10% goat serum (Sigma)/PBS for 1 hour. Thick sections (40-60μm) were permeabilised and blocked in 0.3% triton 10% goat serum/PBS for 3 hours. Primary antibodies were diluted in 10% goat serum/PBS at the indicated concentration (see below) and incubated overnight at 4°C. After washing, the appropriate fluorescent secondary antibody (1/400, Alexa fluor®488, 594 or 647 from

Invitrogen) was used with Hoechst to counterstain the nuclei for 1 hour or 2.5 hours in thin (12μm) and thick (40-60μm) sections respectively. Samples were mounted in Fluoromount G (Southern Biotechnology). To determine the functionality of the blood vessels, fluorescein-conjugated *Griffonia* Simplicifolia lectin I (Vector Lab) was injected into the tail vein 10 minutes prior to harvesting of the animals. The hypoxyprobe-1 kit (hypoxyprobe™) was used to detect hypoxic cells according to the manufacturer's instructions. For HIF-1α immunostaining, nerves were directly embedded in OCT and snap frozen in liquid nitrogen. Cryosections were post-fixed in 4% PFA/PBS, incubated for 15 minutes in 0.1M Glycine/PBS and the above described protocol of immunostaining was performed with an additional tyramide signal amplification step (Invitrogen). For lipocallin-2 immunostaining, 6μm paraffin sections were cut using a microtome (Leica) and wide-field microscopy was used to acquire representative images. For the detection of EdU, nerve sections were stained using the Click-iT® EdU cell proliferation assay kit according to the manufacturer's instructions (Invitrogen).

For immunofluorescence of the fibrin gels, fixation with 4% PFA/PBS was performed for 5 hours at room temperature, followed by permeabilisation of the cells with 0.5% triton/PBS for 2 hours and blocking in 3% BSA/PBS for 5 hours under agitation at room temperature. Primary and secondary antibodies were incubated for 48 hours at 4°C. All fluorescent images were acquired using single photon confocal microscopy (Leica).

Primary antibodies

The following primary antibodies were used for immunofluorescence or for immunohistochemistry staining at the indicated dilutions: p75 (1/500, Millipore ab1554), RECA-1 (1/50, AbD Serotec MCA970), CD31 (1/300, BD Pharmingen 553370), S100 (1/100, Dako Z0311), IBA1 (1/500, Wako 019-19741), mouse-F4/80 (1/500 AbD Serotec MCA497), rat-CD68 (1/100, AbD Serotec MCA5709), prolyl-hydroxylase beta (1/1000, Acris), GFP (1/500, Abcam ab13970), neurofilament 200 kD (1/1000, Abcam ab4680), HIF-1α (1/100, Novus Biologicals NB 100-1231), alkaline-phosphatase-conjugated dioxygenin or AP-DIG (1/1000, Roche), VEGF (1/200 Abcam ab46154), elastin (1/500, Abcam ab21610), fibronectin (1/1000, sigma clone FN-3E2), laminin (1/500, Abcam 11575), collagen I (1/500, Abcam ab292), collagen IV (1/500, Abcam ab19808), paxillin (1/500, Abcam ab32084), talin (1/500, sigma T3287) and beta1 integrin for western (1/500, Abcam ab52971).

Toluidine Blue staining

Sciatic nerves were fixed with 2% glutaraldehyde in 0.2 M phosphate buffer overnight at 4°C, post-fixed in osmium tetroxide for 2 hours at 4°C and then in 2% uranyl acetate for 45 minutes at 4°C. Nerves were then dehydrated in an ethanol series before embedding in

epoxy resin. Semithin sections were cut with a glass knife at 0.1μm, dried carefully and stained with 0.5% toluidine blue in 2% borax at 75°C for 30 seconds. After dehydration, sections were mounted with DPX (Sigma) and representative images were acquired using wide-field microscopy.

Confocal microscopy and image analysis

All fluorescent images were acquired using inverted SPE confocal microscopes (Leica).

For the reconstruction of complete 40-60μm longitudinal sections of sciatic nerves, the section corresponding of the middle of the nerve was selected and images were acquired from the proximal to the distal stump. For each experiment, the same volume and number of z-stacks was taken and the same settings of acquisition were used. For each nerve, a projection of the z-stacks was made using Fiji software (http://fiji.sc/Fiji). The projections were then merged using Adobe Photoshop software.

For blood vessel, Schwann cell and axon area measurements, the reconstructed images were converted to 8-bit grey scale TIFF using Fiji software, the area corresponding to the nerve bridge was then selected, thresholded and made binary. The Create Selection function was used to automatically outline the thresholded area and then the immunostained area was quantified using the measurement function.

For alignment measurements (Figures 7C-E) relative to the nerve proximal to distal axis, NeuronJ was used to draw the cords of Schwann cells or the regrowing axons and Fiji was used to draw the axis of the blood vessels on reconstructed sciatic nerves. The angle of incidence was measured between the proximal to distal nerve axis and blood vessels (300 blood vessels measured per condition from 3 separate animals), Schwann cells (200 cords measured per condition from 3 separate animals) and axons (200 regrowing axons measured per condition from 3 separate animals). For alignment measurements between either Schwann cells and blood vessels or Schwann cells and axons (Figures 7F and S7B), confocal images acquired from the leading edge of migration in the bridge were used to draw the axis of the blood vessels, the cords of Schwann cells and the axons. The angle of incidence was measured between the cell types (100 angles were measured per condition from 3 separate animals of the alignment between Schwann cells and blood vessels and 150 angles were measured from 3 separate animals to determine the alignment of the Schwann cells and axons). In the graphs, each point represents the mean angle for each of the 3 animals +/- SD and the red line represents the mean between the animals for each condition. Rose plots were constructed using Matlab.

Confocal 3-D reconstructions

Figure 3E: Initial reconstruction was carried out in Fiji to identify the blood vessel of interest. The stack was processed to remove background and noise, then cropped and imported into BioVis3D (http://www.biovis3d.com/). Structures were created for each blood vessel interacting with the selected Schwann cell, reconstructed using a quick auto-contour selection tool, then manually adjusted to account for any discrepancies, before creating a 3D surface rendering of a z confocal stack. The nuclei of the selected Schwann cell and the interacting blood vessels were added using manual contour tracing. Snaps were taken at 1024x1024 300dpi at selected angles. Figure 3H: After importation of the LIF file into Imaris software (http://www.bitplane.com/go/products/imaris), the channels corresponding to the blood vessels (blue), the Schwann cells (green) and the axons (red) underwent noise removal. Three surfaces were created, one for each type of structure. Default settings were used in surface wizard, minor adjustments to the intensity threshold were made for Schwann cells and blood vessels to help pick up the dimmer signal. The results were then manually filtered to remove noise in the Schwann cell and axon channels. The images shown in Figure 3H were made using the movie setting on Imaris to calculate the angle and then captured in a snapshot at 1024 x1024 300dpi.

Correlative light and electron microscopy (CLEM)

CLEM – 3D fibrin gel culture: Briefly, GFP-positive Schwann cells in contact with endothelial tubules within the fibrin gel were identified using a fluorescence widefield microscope and imaged together with their location in relation to a photo-etched grid (MatTek Corporation), by fluorescence and brightfield imaging. Cells were then fixed in 4% PFA/PBS overnight and the region of interest was relocated and a razor blade was used to remove all other fibrin gel and cells from the dish to aid infiltration of fixatives and reagents. The samples were then further fixed, dehydrated and embedded in Epon (Stinchcombe et al., 1995). Light microscopy images (xy) were then used to re-locate the GFP-positive Schwann cell in contact with the endothelial tubule in the resin block. For TEM analysis, 70nm sections of the endothelial tubule cut in cross-section (xz) were stained with lead citrate and imaged in a Tecnai 20 (FEI) using a Morada camera and iTEM software (Olympus SIS).

CLEM – 3View: For serial block face imaging, the region of interest was excised, mounted onto a specimen pin and coated with gold palladium before being imaged and sectioned using 3View (Gatan) coupled to a Sigma FEG-SEM (Zeiss). CLEM – ex-vivo: Sciatic nerves from PLP-EGFP mice were collected 10 minutes after tail vein injection of Rhodamine conjugated *Griffonia* Simplicifolia lectin I (Vector Lab), fixed overnight in 4%PFA/PBS at 4°C and embedded in 2.8% low melting point agarose dissolved in L15 media. 100µm thick cross-sections were then cut in cold PBS using a vibrating microtome and serial sections were collected throughout the bridge region of the nerve. Sections were screened using a widefield

fluorescence microscope to identify sections of the nerve bridge where GFP-positive migrating Schwann cells were exiting the stumps. These sections were then imaged at higher magnification using a confocal microscope and full z-stacks were acquired. Sections were then further fixed, dehydrated, and embedded in Epon. Ultrathin sections (70nm) were taken of the whole nerve, and GFP-positive Schwann cells and blood vessels were mapped using both the fluorescence and electron microscopy datasets. More specific regions of interest were identified, and the blocks were further trimmed to allow for collection of serial sections. Serial sections were aligned, cells of interest segmented, rendered and reconstructed in 3D using Amira (FEI).

Supplemental References

Bentzien, F., Zuzow, M., Heald, N., Gibson, A., Shi, Y., Goon, L., Yu, P., Engst, S., Zhang, W., Huang, D., *et al.* (2013). In vitro and in vivo activity of cabozantinib (XL184), an inhibitor of RET, MET, and VEGFR2, in a model of medullary thyroid cancer. Thyroid *23*, 1569-1577.

Gorelik, R., and Gautreau, A. (2014). Quantitative and unbiased analysis of directional persistence in cell migration. Nature protocols *9*, 1931-1943.

Karlsson, G., Rorby, E., Pina, C., Soneji, S., Reckzeh, K., Miharada, K., Karlsson, C., Guo, Y., Fugazza, C., Gupta, R., *et al.* (2013). The tetraspanin CD9 affords high-purity capture of all murine hematopoietic stem cells. Cell Rep *4*, 642-648.

Napoli, I., Noon, L.A., Ribeiro, S., Kerai, A.P., Parrinello, S., Rosenberg, L.H., Collins, M.J., Harrisingh, M.C., White, I.J., Woodhoo, A., *et al.* (2012). A central role for the ERK-signaling pathway in controlling Schwann cell plasticity and peripheral nerve regeneration in vivo. Neuron *73*, 729-742.

Stinchcombe, J.C., Nomoto, H., Cutler, D.F., and Hopkins, C.R. (1995). Anterograde and retrograde traffic between the rough endoplasmic reticulum and the Golgi complex. J Cell Biol *131*, 1387-1401.

Yakes, F.M., Chen, J., Tan, J., Yamaguchi, K., Shi, Y., Yu, P., Qian, F., Chu, F., Bentzien, F., Cancilla, B., *et al.* (2011). Cabozantinib (XL184), a novel MET and VEGFR2 inhibitor, simultaneously suppresses metastasis, angiogenesis, and tumor growth. Mol Cancer Ther *10*, 2298-2308.
