# Supplementary figures and images for: Macrophage-Induced Blood Vessels Guide Schwann Cell-Mediated Regeneration of Peripheral Nerves
Source: Cell. 2015 Aug 27;162(5):1127–39. doi: 10.1016/j.cell.2015.07.021 (PMC4553238; doi:10.1016/j.cell.2015.07.021)

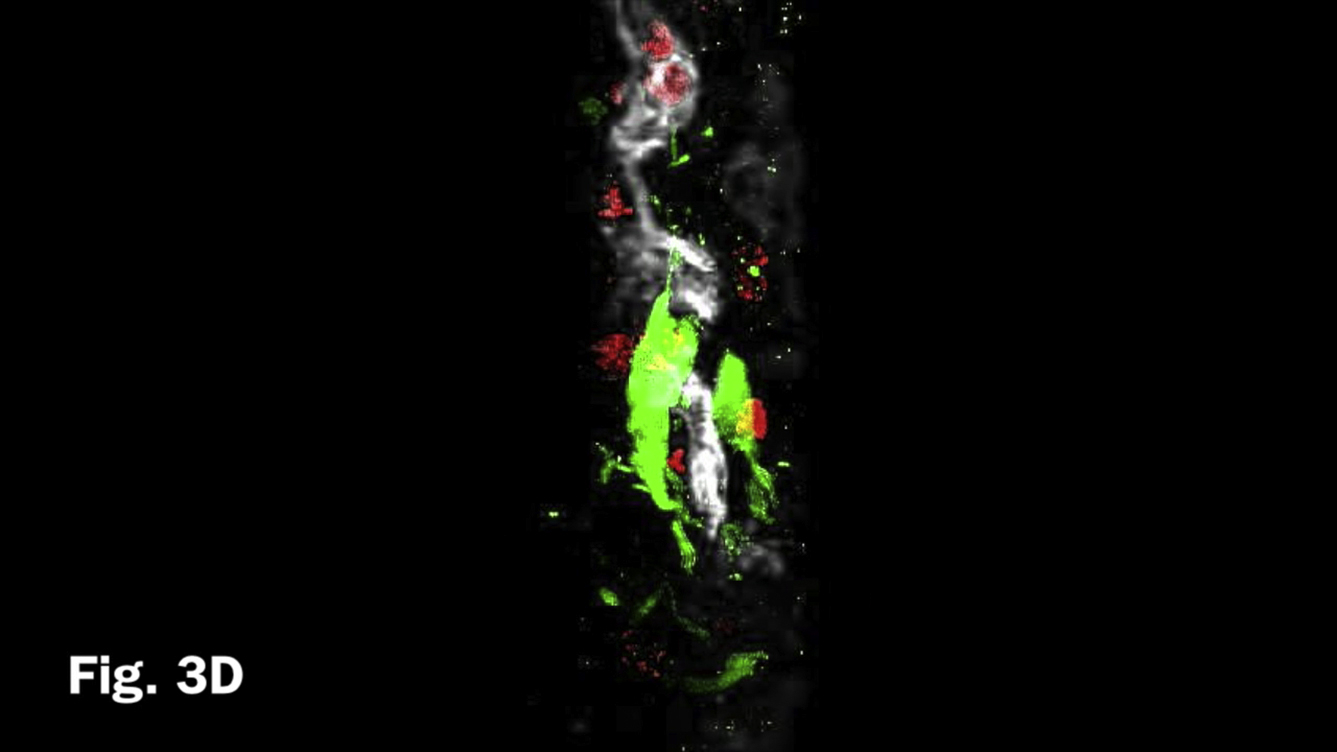

Supplement: Movie S1. In Vivo 3D Reconstruction of Confocal Images Showing Schwann Cells Migrating along the Vasculature, Related to Figure 3 — Rotating 3D projection showing a S100-positive Schwann cell (green) interacting with an EdU-positive (red) blood vessel (white) as shown in Figure 3D. Z stacks of confocal images were processed using Fiji software. Rotating 3D reconstruction showing GFP-positive migrating cords of Schwann cells and their associated axons interacting with the vasculature within the nerve bridge as shown in Figure 3H. Z stacks of confocal images of longitudinal sections from an injured sciatic nerve of PLP-EGFP mice stained for CD31 (blue) and neurofilament (NF, red) were processed using the Imaris software to make a 3D surface-rendered reconstruction of the interaction between the vasculature and Schwann cells. [file mmc2.jpg]

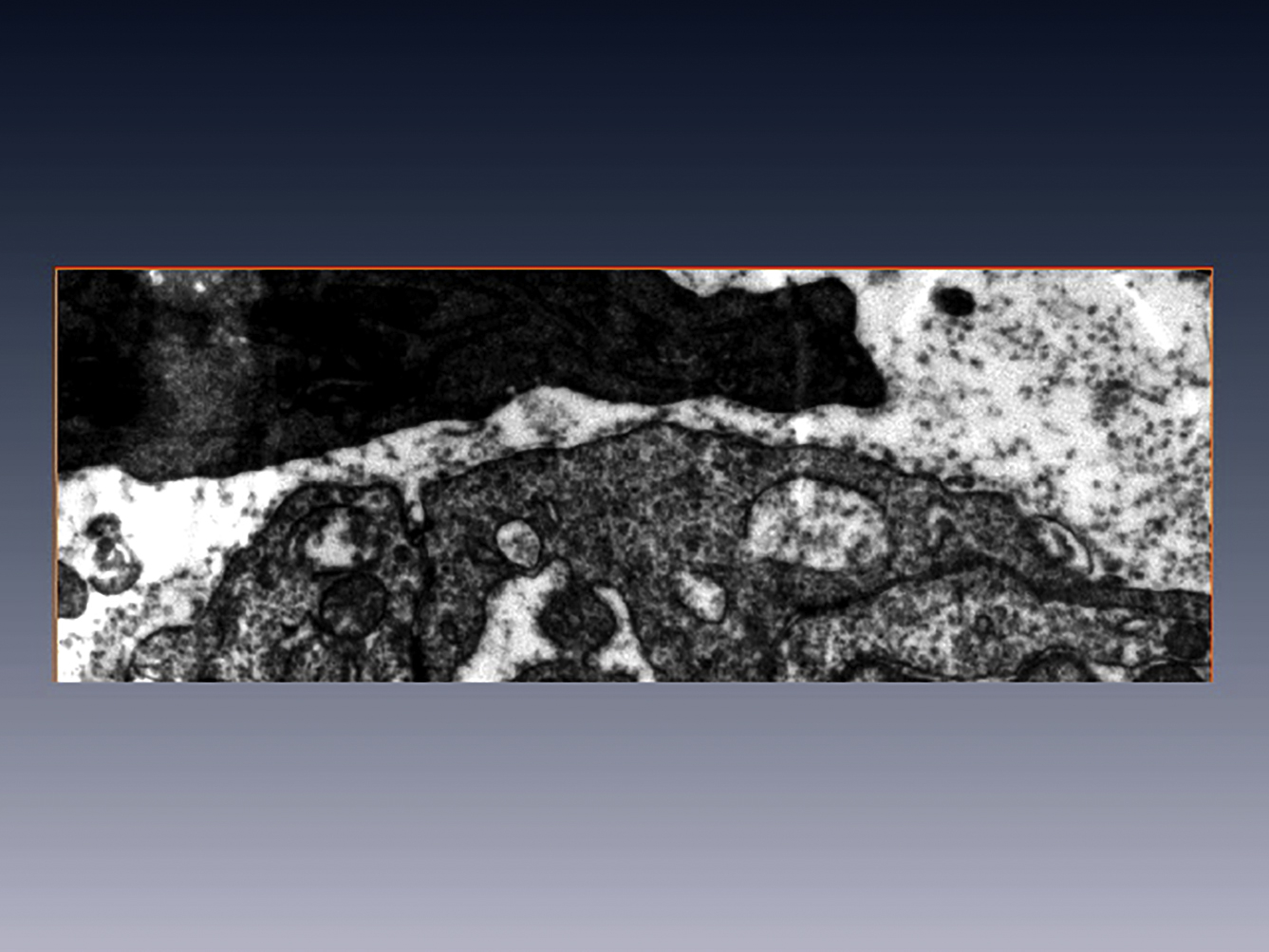

Supplement: Movie S2. 3D Reconstruction of TEM Images Showing the Points of Contact between a Migrating Schwann Cell and a Blood Vessel within the Bridge Region of a Regenerating Nerve, Related to Figure 3 — 3D reconstruction showing direct contact between a migrating Schwann cell (green) and an endothelial cell (yellow) in vivo as shown in Figures 3G and S3D. Serial 70 nm sections were imaged, aligned, segmented and rendered in Amira to produce a 3D reconstruction of the contact between a Schwann cell (green) and an endothelial cell (yellow) identified by correlative light and electron microscopy of a 100 μm vibrotome section of an injured sciatic nerve from a PLP- EGFP mouse. [file mmc3.jpg]

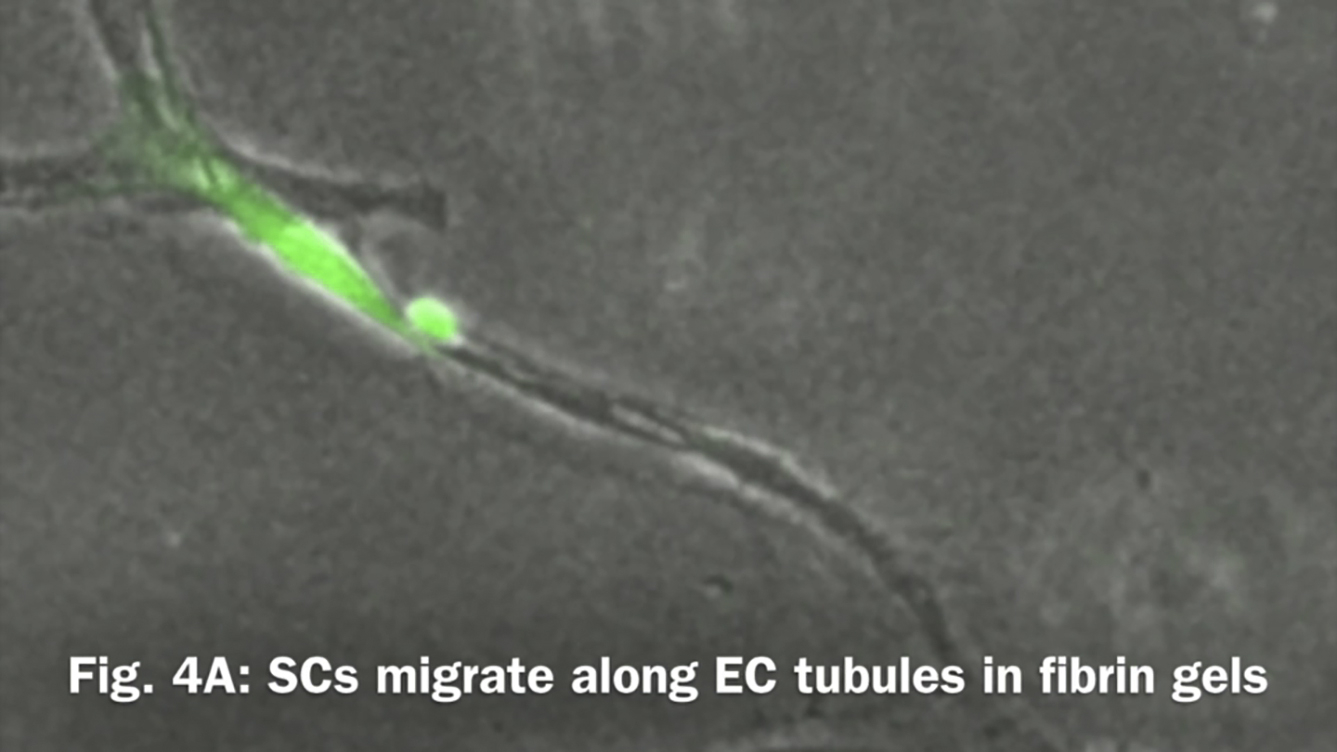

Supplement: Movie S3. In Vitro Migration of Schwann Cells along Endothelial Cell Tubules, Related to Figure 4 — Time-lapse microscopy of a GFP-expressing rat Schwann cell migrating along a tubule of HUVECs within a fibrin gel as shown in Figure 4A. Frames were taken every 10 min for 10 hr. GFP fluorescence and phase contrast are shown. Time-lapse microscopy of a GFP-expressing rat Schwann cells migrating along a tubule of HUVECs within a fibrin gel. Frames were taken every 10 min for 15 hr. GFP fluorescence and phase-contrast are shown. Time-lapse microscopy of a GFP-expressing rat Schwann cell within a fibrin gel. Frames were taken every 10 min for 10 hr. GFP fluorescence and phase-contrast are shown. Time-lapse microscopy of GFP-expressing rat Schwann cells migrating along a tubule of HUVECs in Matrigel. Frames were taken every 10 min for 8 hr. GFP fluorescence and phase-contrast are shown initially followed by the same movie showing only GFP fluorescence, in order to observe more clearly the migratory behavior of the Schwann cell. [file mmc4.jpg]

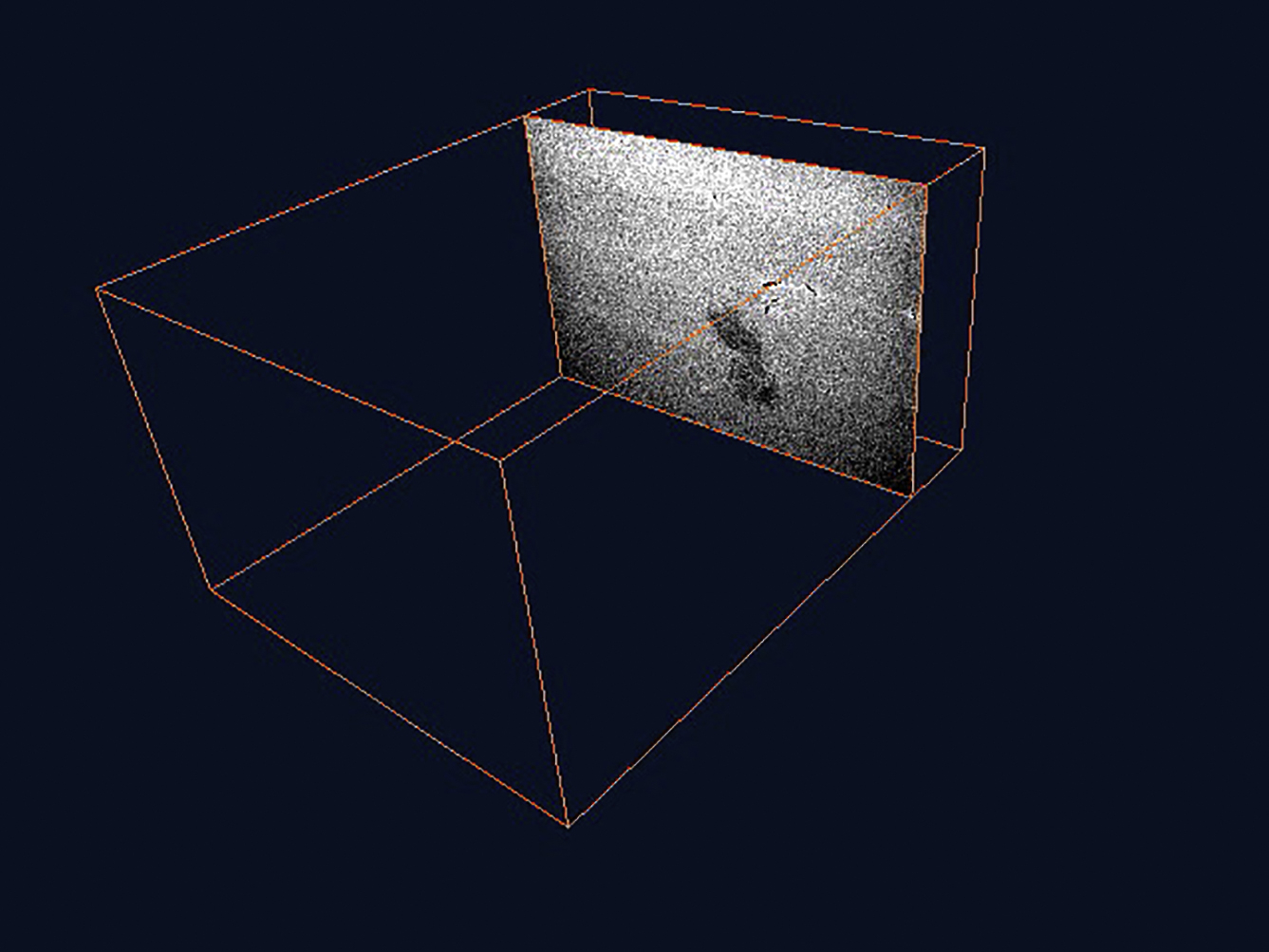

Supplement: Movie S4. 3D Reconstruction of Serial Block Face Images Showing the Contact between a Migrating Schwann Cell and an Endothelial Cell Tubule, Related to Figure 4 — 3D-reconstruction showing direct contact between a migrating Schwann cell (green) and a tubule of HUVECs (red) within a fibrin gel as shown in Figure 4B. After serial block face imaging of the tubule using a Sigma FEG-SEM coupled to a 3View, images were processed using Amira software to generate a 3D-reconstruction of the contact between the Schwann cell and the endothelial cells. [file mmc5.jpg]

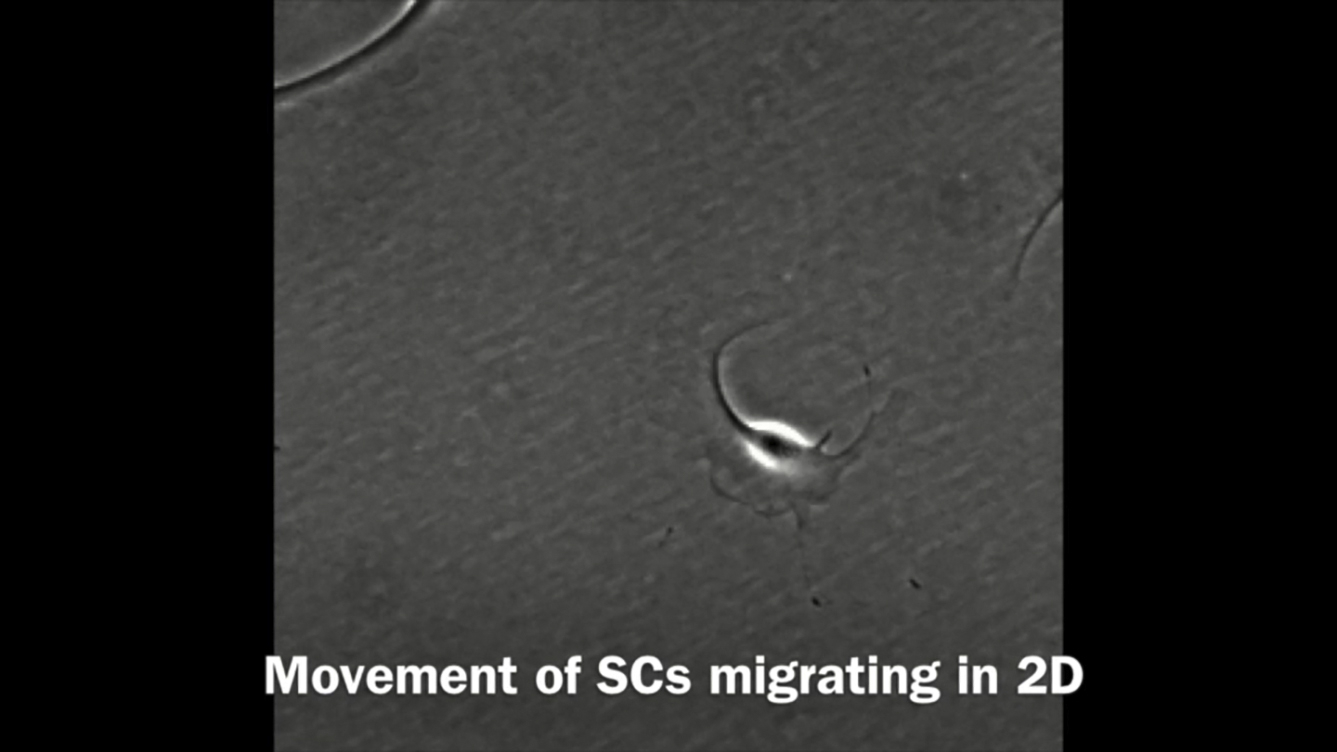

Supplement: Movie S5. Mode of Migration of Schwann Cells in 2D versus 3D, Related to Figure 4 — Time-lapse microscopy of GFP-expressing rat Schwann cells migrating on a 2D laminin-coated surface. Frames were taken every 10 min for 10 hr. Phase-contrast is shown. Time-lapse microscopy to exemplify the mode of migration of a GFP-expressing rat Schwann cell migrating along a tubule of HUVECs within a 3D fibrin gel. GFP fluorescence and phase-contrast are shown initially followed by the same movie showing only GFP fluorescence, in order to observe more clearly the migratory behavior of the Schwann cell. Frames were taken every 10 min for 10 hr. See snapshots in Figure 4D. Time-lapse microscopy showing a GFP-expressing rat Schwann cell at a higher magnification migrating along a tubule of HUVECs within a fibrin gel. Frames were taken every 10 min for 8 hr. GFP fluorescence and phase-contrast are shown initially followed by the same movie showing only GFP fluorescence, in order to observe more clearly the migratory behavior of the Schwann cell. [file mmc6.jpg]

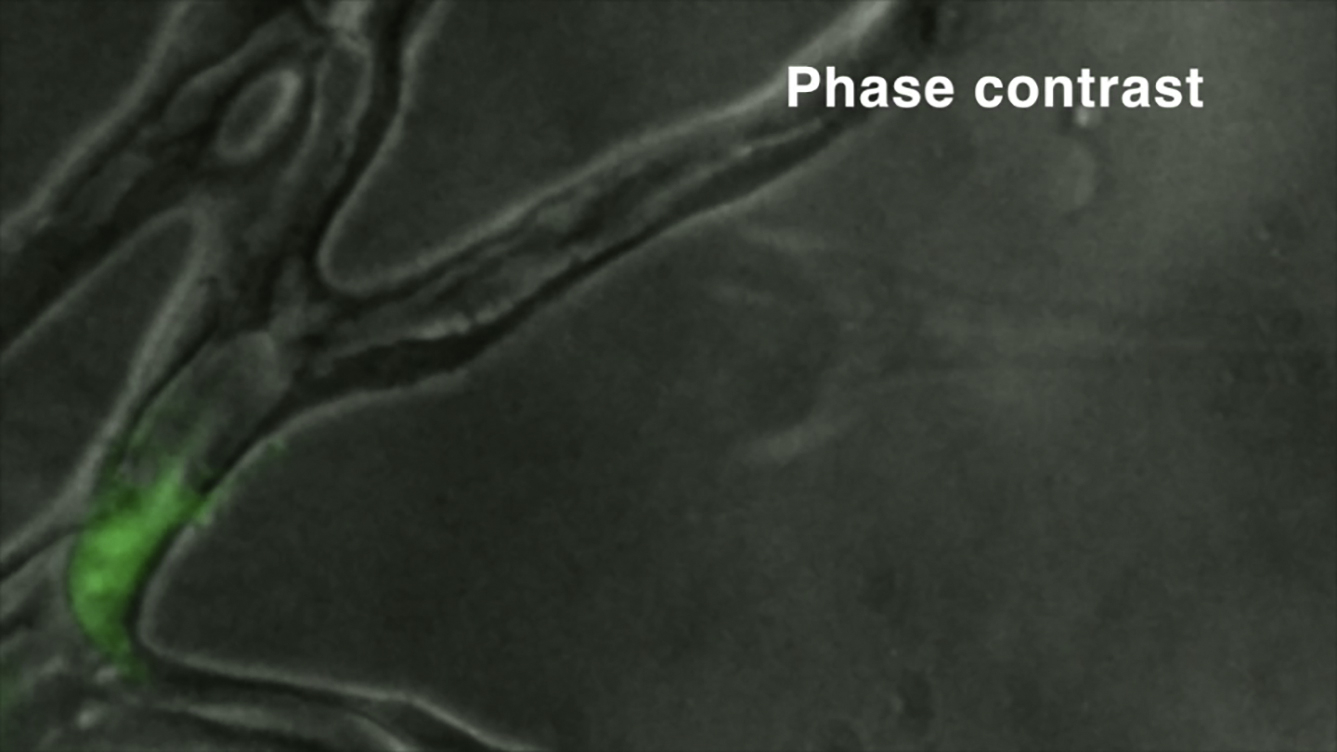

Supplement: Movie S6. Schwann Cell Migration along Blood Vessels Is Dependent on Rear Actomyosin Contractility, Related to Figure 4 — Time-lapse microscopy of a GFP-expressing rat Schwann cell migrating along a tubule of HUVECs within a fibrin gel. Frames were taken every 7.5 min for 10 hr. The Rho-kinase inhibitor (Y-27632 50 μM) was added after 5 hr. GFP fluorescence and phase-contrast are shown initially followed by the same movie showing only GFP fluorescence. Results are quantified in Figure S4G. Time-lapse microscopy of a GFP-expressing rat Schwann cells migrating along a tubule of HUVECs within a fibrin gel. Frames were taken every 10 min for 7.5 hr, latrunculin B (0.2 μM) was added after 5 hr. GFP fluorescence and phase-contrast are shown initially followed by the same movie showing only GFP fluorescence. Results are quantified in Figure S4G. [file mmc7.jpg]

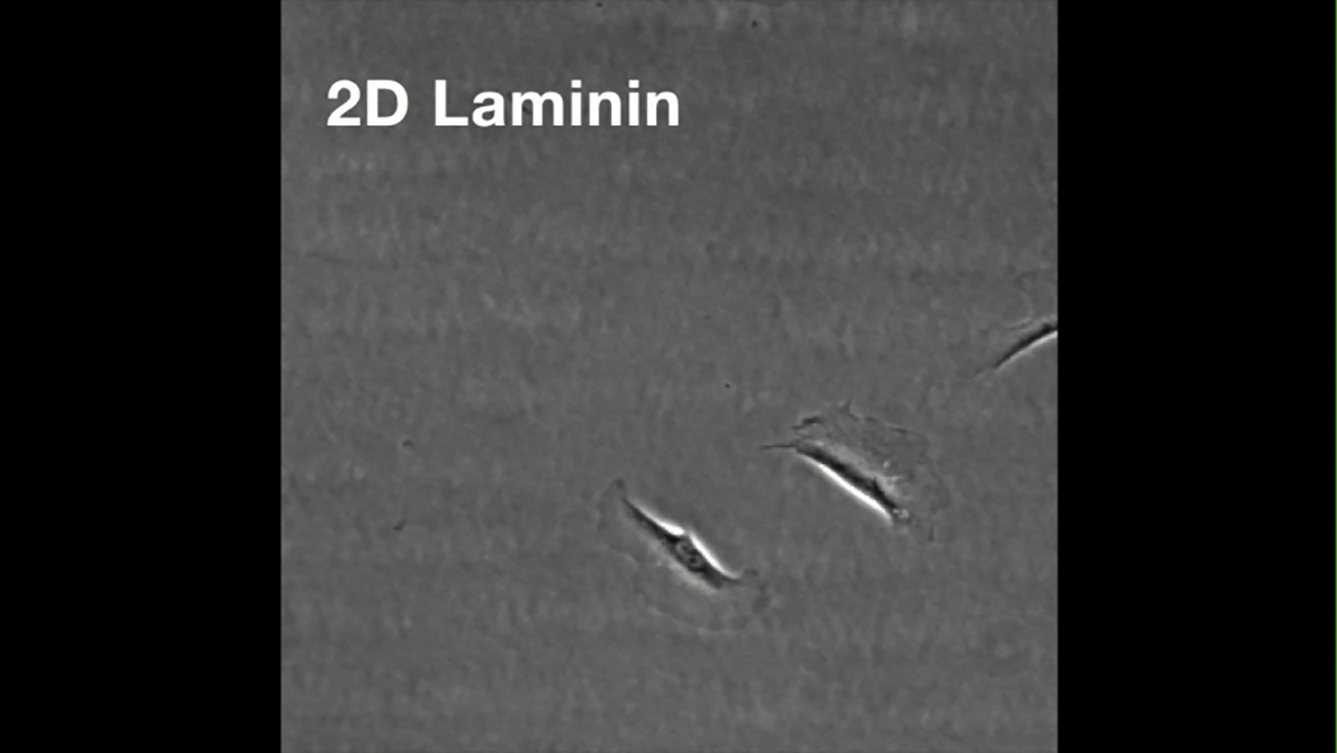

Supplement: Movie S7. Schwann Cell Migration along Blood Vessels Is Independent of beta1 Integrin, Related to Figure 4 — Time-lapse microscopy of beta1 integrin knocked down GFP-expressing rat Schwann cells migrating on a 2D laminin-coated surface followed by time-lapse microscopy of a cell from the same experiment migrating along tubules of HUVECs in Matrigel. Frames were taken every 10 min for 4 hr. Phase contrast is shown for the cells migrating on laminin and both GFP fluorescence and phase contrast are shown for the Matrigel experiments. Note the migration of the cells is severely inhibited in 2D but is unaffected in 3D. Results are quantified in Figure S4I. [file mmc8.jpg]
