# Supplementary material for: Macrophage-Induced Blood Vessels Guide Schwann Cell-Mediated Regeneration of Peripheral Nerves
Source: Cell. 2015 Aug 27;162(5):1127–39. doi: 10.1016/j.cell.2015.07.021 (PMC4553238; doi:10.1016/j.cell.2015.07.021)
Supplement: Document S2. Article plus Supplemental Information [file mmc9.pdf]

Cell

Article

Macrophage-Induced Blood Vessels Guide Schwann Cell-Mediated Regeneration of Peripheral Nerves

Graphical Abstract

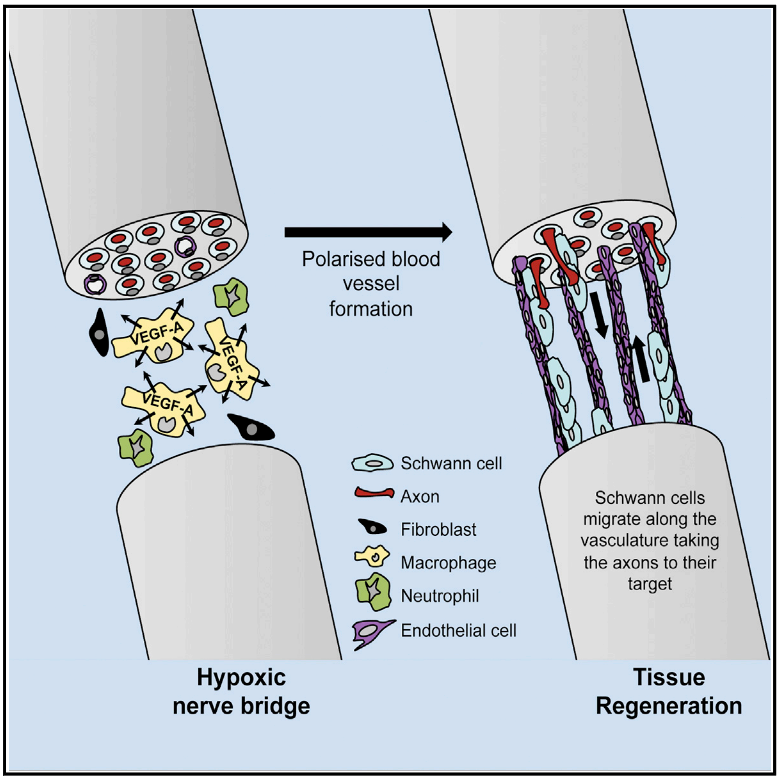

Authors

Anne-Laure Cattin, Jemima J. Burden, Lucie Van Emmenis, ..., Tariq Enver, Christiana Ruhrberg, Alison C. Lloyd

Correspondence

alison.lloyd@ucl.ac.uk

In Brief

Repairing a cut nerve requires collective migration of Schwann cells guided by a polarized vasculature that is induced by macrophages within the hypoxic bridge.

Highlights

- Hypoxia within the nerve bridge is selectively sensed by macrophages
- Macrophage-derived VEGF-A induces a polarized vasculature within the bridge
- Blood vessels are used as tracks to direct Schwann cell migration across the wound
- Macrophage-induced blood vessels are essential for nerve regeneration

Cattin et al., 2015, Cell *162*, 1127–1139
August 27, 2015 ©2015 The Authors
http://dx.doi.org/10.1016/j.cell.2015.07.021

Cell

Macrophage-Induced Blood Vessels Guide Schwann Cell-Mediated Regeneration of Peripheral Nerves

Anne-Laure Cattin,[1] Jemima J. Burden,[1] Lucie Van Emmenis,[1] Francesca E. Mackenzie,[3] Julian J.A. Hoving,[1] Noelia Garcia Calavia,[1] Yanping Guo,[2] Maeve McLaughlin,[1] Laura H. Rosenberg,[1] Victor Quereda,[1] Denisa Jamecna,[1] Ilaria Napoli,[1] Simona Parrinello,[1] Tariq Enver,[2] Christiana Ruhrberg,[3] and Alison C. Lloyd[1,2,*]
[1]MRC Laboratory for Molecular Cell Biology, UCL, Gower Street, London WC1E 6BT, UK
[2]UCL Cancer Institute, UCL, 72 Huntley Street, London WC1E 6DD, UK
[3]Department of Cell Biology, UCL Institute of Ophthalmology, 11-43 Bath Street, London EC1V 9EL, UK
*Correspondence: alison.lloyd@ucl.ac.uk
http://dx.doi.org/10.1016/j.cell.2015.07.021
This is an open access article under the CC BY license (http://creativecommons.org/licenses/by/4.0/).

SUMMARY

The peripheral nervous system has remarkable regenerative capacities in that it can repair a fully cut nerve. This requires Schwann cells to migrate collectively to guide regrowing axons across a 'bridge' of new tissue, which forms to reconnect a severed nerve. Here we show that blood vessels direct the migrating cords of Schwann cells. This multicellular process is initiated by hypoxia, selectively sensed by macrophages within the bridge, which via VEGF-A secretion induce a polarized vasculature that relieves the hypoxia. Schwann cells then use the blood vessels as "tracks" to cross the bridge taking regrowing axons with them. Importantly, disrupting the organization of the newly formed blood vessels in vivo, either by inhibiting the angiogenic signal or by re-orienting them, compromises Schwann cell directionality resulting in defective nerve repair. This study provides important insights into how the choreography of multiple cell-types is required for the regeneration of an adult tissue.

INTRODUCTION

The creation of tissues during development requires the temporal coordination of multiple cell types by a combination of intrinsic and extrinsic developmental signals that control the number and movement of cells (Bryant and Mostov, 2008; Martin and Parkhurst, 2004). Few tissues in the adult mammal are able to recapitulate these processes to regenerate following injury; in some cases, this is due to the absence in the adult of the stem cells that originally gave rise to the tissue, however, the absence of extrinsic developmental, morphogenic and guidance cues present in the developing organism is also likely to play a major role (Poss, 2010). The peripheral nervous system (PNS) is one tissue able to regenerate in the adult mammal. This is all the more remarkable because of the complex structure of nerves and that regeneration requires the regrowth and coordination of multiple cell types over long distances within the architecture of the adult tissue (Zochodne, 2008).

Peripheral nerves consist of bundles of axons, with each axon associated and enveloped by Schwann cells (SCs), the main glial cell of the PNS. SCs either exist in a 1:1 ratio with larger diameter axons, which they myelinate, or group together smaller axons in structures known as Remak bundles. Groups of these axons are further organized into a fascicle, enclosed by the perineurium, which is made-up of layers of specialized, fibroblast-like cells. Several fascicles can be further enclosed within the epineurial sheath that surrounds each nerve. The axons exist in a specialized, privileged compartment, known as the endoneurium, protected by the blood/nerve barrier, which is maintained by both the perineurium and by specialized blood vessels that run throughout the nerve. Fibroblasts and macrophages also reside within the matrix of this compartment (Zochodne, 2008).

Remarkably, in contrast to nerves in the CNS, peripheral nerves can regenerate even following a complete transection. Following a transection, the stumps retract and in the distal part of the nerve, the axons, separated from their cell bodies, rapidly degenerate by an active process known as Wallerian degeneration (Zochodne, 2008). The major aim of the regeneration process is for the axons to regrow back to their targets, which requires guidance signals distinct from those that originally directed the axons during development (Dudanova and Klein, 2013). Following an injury, the SCs in both the proximal stump and throughout the nerve downstream of the cut dedifferentiate to a progenitor-like cell, which proliferate, orchestrate an inflammatory response that clears the debris and remodels the environment (Napoli et al., 2012). In the distal stump, these cells form tube-like structures within their original basement membranes, known as bands of Büngner, which can act as "tunnels" to direct the regrowing axons back to their original targets. However, following a transection, the basement membranes are broken and distinct mechanisms are required to direct the regrowing axons into the bands of Büngner in the distal stump (Fawcett and Keynes, 1990; Nguyen et al., 2002).

By an unknown mechanism, following transection, the two stumps are rejoined by a poorly characterized structure known as "the bridge" that can be several millimeters in length and is composed of a mixture of inflammatory cells and matrix (Jurecka et al., 1975) and thus seemingly a hostile and non-directional

environment for axonal regrowth. We recently showed that SCs are responsible for guiding the axons across this bridge region (Parrinello et al., 2010). This contrasts to during development, when axons are guided to their targets by a combination of extrinsic attractive and repulsive signals (Dudanova and Klein, 2013), with the SCs following behind the axons on this journey (Heermann and Schwab, 2013). We showed that cords of SCs migrate out of both distal and proximal stumps until they extend across the bridge, with SC cords from the proximal stump taking the regrowing axons with them. This organized migration is directed by fibroblasts at the wound site that, via EphrinB/EphB2 signaling, convert normally repulsive SCs to an adhesive behavior necessary for their collective migration. Importantly, loss of this signal results in disruption of the SC cords and loss of the directional movement that directs the axons toward the distal stump (Parrinello et al., 2010). However, while these studies showed that the cords of SCs were critical for the directional growth of the axons, it remained unclear how the SCs themselves were finding their way across the bridge.

In this study, we identify critical roles for both chemotactic cues from within the bridge, as well as the topography of the bridge in directing SC migration during PNS regeneration. We find that hypoxia within the bridge is selectively responded to by macrophages, which via VEGF-A secretion triggers the polarized vascularization of the bridge region. These newly formed blood vessels are subsequently used as a guiding path by SCs to invade and cross the bridge, taking the regrowing axons with them. Importantly, we show that the blood vessels are necessary and sufficient to guide the migration of SCs, as misdirection of the blood vessels leads the SCs into surrounding tissues. This work describes how mechanisms distinct from those used during development can regenerate a tissue in the adult. Moreover, it identifies an unanticipated role for macrophage-induced blood vessels in this process that has implications for improving the regenerative process following injury and provides insights into the way cells may spread in pathologies such as cancer.

RESULTS

Polarized Blood Vessels Form within the Bridge prior to Schwann Cell Migration

In order to determine the mechanism by which SC cords are directed across the bridge, we first determined the cell composition of the bridge, prior to SC migration. In the vast majority of rats (>95%), a bridge between the distal and proximal nerve stumps was formed 2 days following transection (day 2). In agreement with previous observations (Avellino et al., 1995; Jurecka et al., 1975), we found that the bridge was mostly composed of macrophages (50%) and neutrophils (24%) with fibroblasts (13%) and endothelial cells (ECs) (5%) as minor components (Figures 1A and S1A). At day 3, however, we noted a significant increase in the number of ECs indicating that the bridge may have become vascularized (Figure 1A). Consistent with this, vascularization could be observed macroscopically (Figure S1B) and confocal analysis of the entire bridge region confirmed this to be the case; at day 2, the bridge contained very few blood vessels whereas by day 3, there was a dramatic influx of blood vessels, which permeated the entire bridge (Figure 1B, quantified in Figures 1C and 1D). Analysis of nerves in which the vascularization was at a slightly earlier stage showed that the blood vessels appeared to emanate from both proximal and distal stumps (Figure S1C) but importantly, the nerve was fully vascularized by the time the SC cords entered into the bridge from either stump (Figures 1B and S1D). In mice, the regeneration process is similar but takes place more slowly, but as observed in rats, we found that the bridge was fully vascularized prior to SC migration into the bridge (Figures 1E and S1D). Thus in both rats and mice, ECs cross the bridge prior to the migration of the SC cords.

Analysis of the incorporation of EdU found that all the blood vessels within the bridge contained EdU-positive ECs, confirming that they were newly formed (Figures 2A, 2B, and S2A). In contrast, EdU-positive ECs were not found within uncut nerve (Figures 2A and 2B) or in the proximal or distal stumps (Figure S2B). Moreover, erythrocyte staining and the detection of injected immunolabeled-lectin showed that the majority of the newly formed blood vessels within the bridge were functional (Figures S2C and S2D).

Remarkably, the blood vessels within the bridge of both rats and mice appeared to be similarly organized, in that the majority seemed aligned to the direction that the SC cords would subsequently travel across the bridge (Figures 2C and 2D). Quantification of longitudinal sections of the bridges established that ~80% of the blood vessels were orientated in the direction of subsequent SC migration (Figures 2E and 2F). This polarized growth of blood vessels prior to SC migration raised the possibility that blood vessels provide directional signals to the cords of SCs to help them find their way across the bridge.

Schwann Cells Interact Directly with Polarized Blood Vessels

Confocal microscopy analysis of the SCs entering into the bridge demonstrated a close association of the migrating SC cords and the polarized blood vessels (Figure 3A). Moreover, at later time points when the SCs had migrated further into the bridge, these interactions were maintained (Figure 3B). Analysis of matrix components of the bridge showed that fibronectin filled the space between the cells throughout the bridge and that strands of elastin also permeated the bridge region. In contrast, laminin and collagen I and IV could be detected only around the blood vessels (Figure S3A). To quantify the degree and specificity of the interactions between the SCs and the blood vessels, we measured the shortest distance between the nuclei of SCs at the leading edge and their closest blood vessel and compared it to the distance of other cell types present in the bridge. We found that the majority of SCs were extremely close (<10 μm) to blood vessels with the population showing a strong distribution toward the blood vessels, whereas the other cell types had a more random distribution within the bridge (Figure 3C). Moreover, the degree of interaction between SCs and blood vessels was probably underestimated, as we frequently observed SCs interacting with blood vessels via long protrusions while the nuclei were further away.

Higher resolution, 3D-projection views of the bridge indicated that SCs were making direct physical contacts with the EdU-labeled vasculature (Figure 3D; Movie S1). Moreover, 3D surface

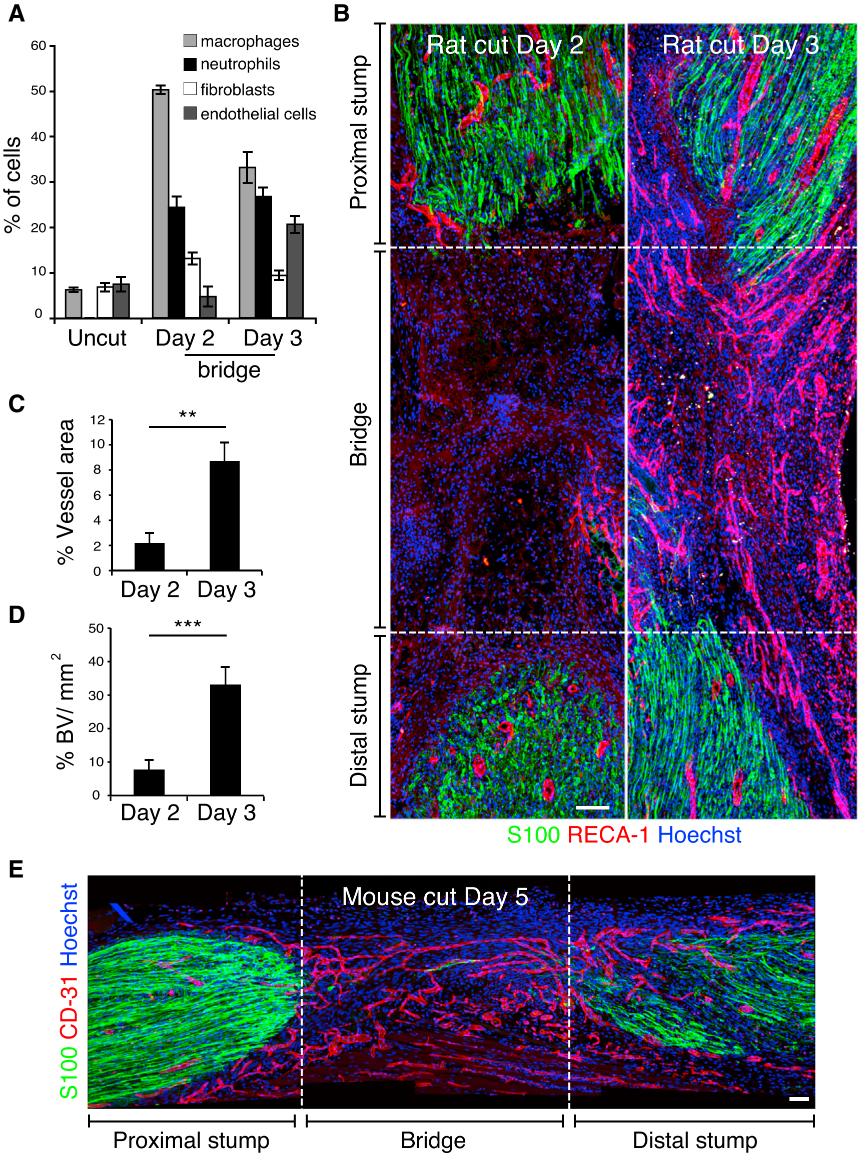

Figure 1. Blood Vessels Permeate the Bridge prior to SC Migration

(A) Graph shows the proportion of macrophages (Iba1$^+$), fibroblasts (prolylhydroxylase$^+$/ Iba1$^-$), ECs (RECA-1$^+$), and neutrophils (lipocallin-2$^+$) within the bridge of transected rat sciatic nerves and in contralateral intact nerves (Uncut), Day 2, and Day 3 after transection (n = 4, graph shows mean value ± SEM).

(B) Rat sciatic nerve longitudinal sections immunostained for ECs (RECA-1$^+$, red) and SCs (S100$^+$, green), Day 2 and Day 3 after transection. Nuclei were counterstained with Hoechst (blue). Scale bar, 100 μm.

(C and D) Quantification of the vascularization of the bridge as shown in (B). (C) Graph shows the percentage of RECA-1 positive area at the indicated times (n = 6). (D) Graph shows the average number of blood vessels/mm^2 of bridge at the indicated times (n = 6). Graphs show mean value ± SEM.

(E) Longitudinal section of a mouse sciatic nerve immunostained for ECs (CD31$^+$, red) and SCs (S100$^+$, green), Day 5 after transection. Scale bar, 100 μm. For reconstruction of longitudinal sections shown in (B) and (E), multiple images from the same sample were acquired using the same microscope settings.

See also Figure S1.

rendering of high-resolution confocal z-stacks of the bridge confirmed the presence of multiple physical contacts between SCs and the surrounding blood vessels (Figure 3E).

To analyze the interactions between the migrating SC cords and the blood vessels in the mouse, we used a transgenic mouse in which EGFP is specifically expressed in SCs (PLP-EGFP) (Mallon et al., 2002). Co-immunostaining of the vasculature showed GFP-positive migrating SCs closely associated with the blood vessels in the bridge (Figure 3F). EM analysis of the blood vessels showed that compared to established vessels within the contralateral nerve, the vessels of the bridge had an extremely thin basal lamina with regions where there appeared to be little or no matrix that could allow direct contact between the cells (Figures S3B and S3C). Moreover, using correlative light and electron microscopy (CLEM) of blood vessels and SCs within the bridge, we could observe multiple points of direct contact between the two cell types (Figures 3G and S3D). Interestingly, 3D reconstruction of serial sections along the blood vessel showed that the interactions occurred between blebs and protrusions emanating from both cell types, providing a discontinuous surface that could potentially generate traction for movement (Figure 3G; Movie S2) (Bergert et al., 2015; Liu et al., 2015; Tozluoğlu et al., 2013).

3D surface rendering of high-resolution confocal z-stacks of the bridge co-stained with an axonal marker confirmed the direct interaction between the cords of SCs and the blood vessels and showed the SC cords guiding the axons along them (Figure 3H; Movie S1). Thus, in both rats and mice, SCs physically interact with polarized blood vessels as they migrate and guide axons across the bridge.

Schwann Cells Migrate along Capillary-like Endothelial Cells In Vitro

To test whether SCs could interact directly with blood vessels in a simplified system, we co-cultured GFP-positive rat SCs with human umbilical vein endothelial cells (HUVECs), which had been coated onto beads and then placed into a fibrin matrix to form capillary-like structures (Nakatsu et al., 2003). Time-lapse microscopy showed that the vast majority of SCs interacted with the endothelial cell tubules and migrated along them (Figures 4A and S4A; Movie S3). Importantly, confocal microscopy images confirmed that the migrating SCs made direct physical

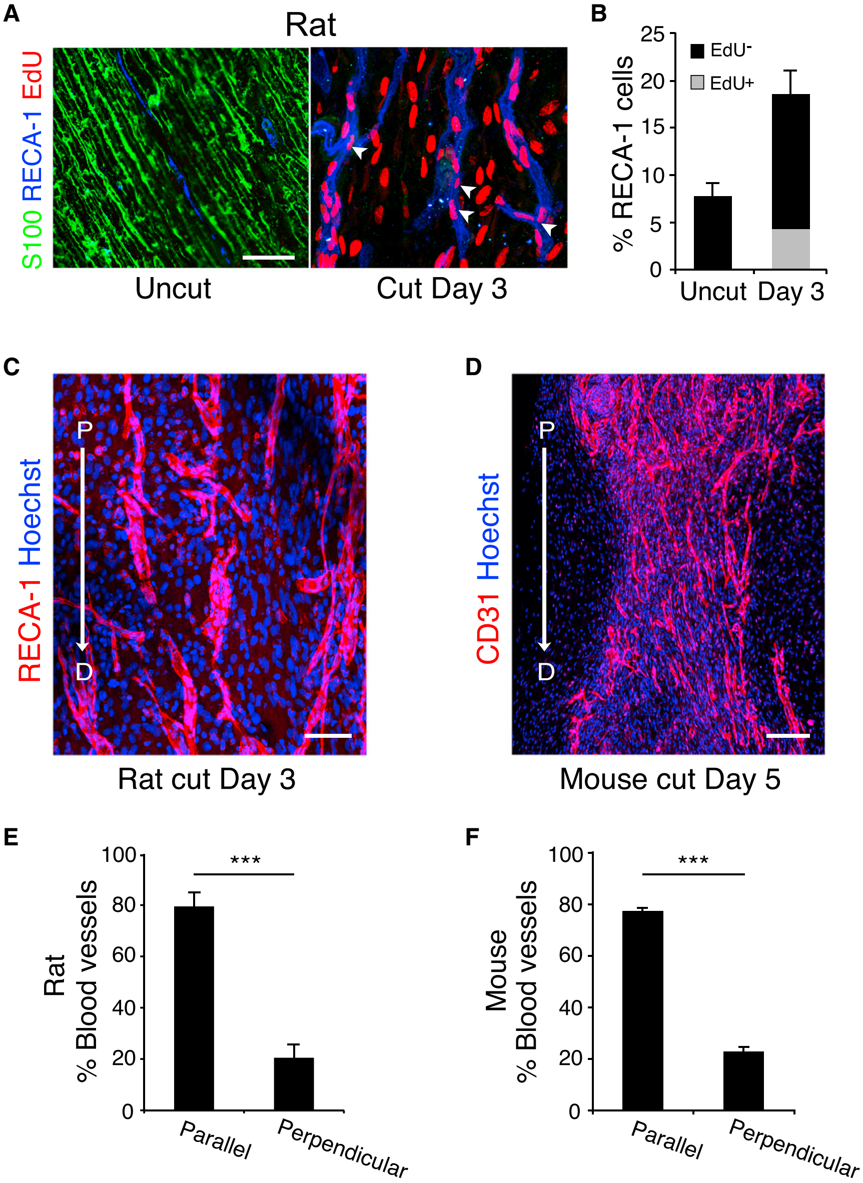

Figure 2. Newly Formed Blood Vessels in the Bridge Are Polarized in the Direction of SC Migration

(A) Representative longitudinal sections of a rat sciatic nerve bridge and the contralateral uninjured nerve, day 3 after transection, and 12 hr after EdU injection. EdU^+ cells (red) were co-labeled to detect ECs (blue) and S100 to detect SCs (green). Scale bar, 25 μm. White arrowheads indicate EdU^+ ECs.

(B) Quantification of the proportion of EdU^+ ECs in the bridge, day 3 after transection compared to uncut (n = 4).

(C) Representative confocal image of a longitudinal section of a rat nerve bridge immunostained for ECs ($RECA\text{-}1^+$) at day 3 after transection. Scale bar, 50 μm. Arrow indicates the direction of axonal growth from the proximal (P) to the distal (D) stump.

(D) Representative confocal image of a longitudinal section of a mouse nerve bridge immunostained for ECs ($CD31^+$) at Day 5 after transection. Scale bar, 100 μm. For reconstruction of longitudinal sections shown in (C) and (D), multiple images from the same sample were acquired using the same microscope settings.

(E and F) Quantification of the proportion of blood vessels parallel or perpendicular to the direction of SC migration in the rat bridge (E) or the mouse bridge (F) (n = 4).

Graphs show mean value ± SEM. See also Figure S2.

contacts with the ECs as observed in vivo (Figure S4B). A small proportion of the SCs remained within the matrix (<15%) (Figure S4A), yet while these cells were able to form protrusions they were unable to migrate efficiently (Movie S3). In contrast, and consistent with other studies (Hakkinen et al., 2011), we found that fibroblasts, when added to the matrix, did not interact specifically with the tubules but instead spread and migrated within the matrix (Figure S4C). We confirmed that SCs directly migrated along blood vessels by generating tubules of HUVECs in a second matrix, Matrigel and found that SCs migrated efficiently along them (Movie S3). This demonstrates that SCs, unlike fibroblasts, are unable to migrate efficiently within a 3D matrix unless they associate with a scaffold of EC tubules.

To analyze the nature of the interaction between SCs and ECs in vitro, we performed CLEM on GFP-positive SCs interacting with ECs within the fibrin gel. We observed that SCs made direct contact with the ECs, with serial block face imaging demonstrating contacts along the length of the migrating SC (Figure 4B; Movie S4). Higher resolution TEM analysis revealed that the interactions were distinct from those between neighboring ECs, which formed a tight uniform junction, consistent with a stable interaction between the cells. In contrast, the contacts between the SCs and ECs although direct, occurred at multiple discrete sites, a morphology consistent with the dynamic movement of the SC along the EC tubules and consistent with our in vivo findings (Figure 4C).

To study further the mode of migration, we analyzed multiple time-lapse movies and compared to SC migration in 2D. The SCs moved faster in 3D and showed a greater persistence of direction as they migrated along the blood vessels (Figures S4D–S4F). In 2D, SCs move in a classical adhesion-dependent manner involving large lamellipodia-like structures (Movie S5). In contrast in 3D, the SCs exhibited a more ameboid-like mode of migration with the extension of protrusions followed by a contraction of the rear of the cell, a movement characteristic of conditions of lower adhesion and higher levels of confinement (Figure 4D; Movie S5) (Lämmermann and Sixt, 2009; Liu et al., 2015). Consistent with this, we found that the rear-contraction

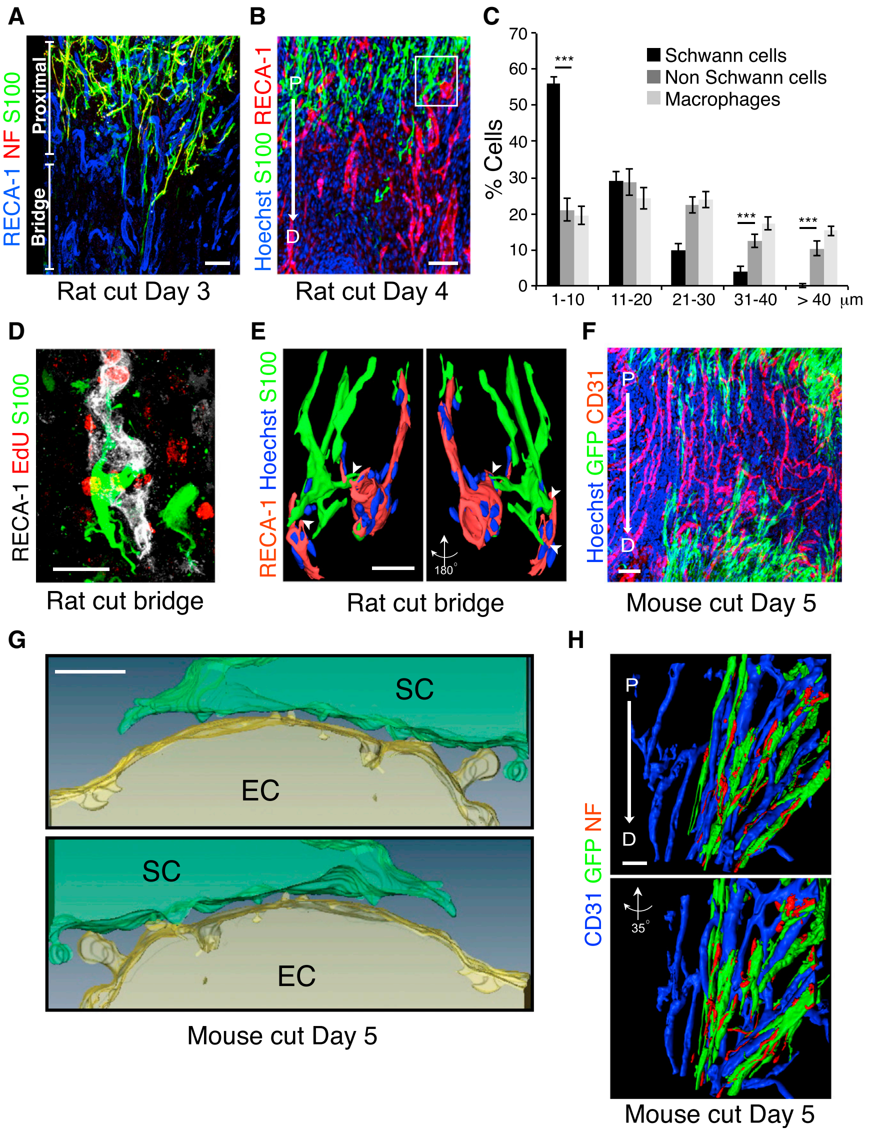

Figure 3. Migrating SCs Interact with the Vasculature of the Bridge

(A) Representative confocal image of a longitudinal section of a rat sciatic nerve bridge, Day 3 after transection, immunostained to detect axons (neurofilament (NF), red), SCs ($S100^+$, green), and ECs ($RECA\text{-}1^+$, blue) and shows cords of SCs and associated regrowing axons interacting with the vasculature as they emerge from the proximal stump and enter the bridge. Scale bar, 50 µm.

(B) Rat sciatic nerve longitudinal sections immunostained to detect SCs ($S100^+$, green) and ECs ($RECA\text{-}1^+$, red), Day 4 after transection. Scale bar, 100 µm. White rectangle indicates the region used to build the 3D model shown in (E). For reconstruction of longitudinal sections shown in (A) and (B), multiple images from the same sample were acquired using the same microscope settings.

(C) Frequency distribution graph showing the distance of the nuclei of SCs ($S100^+$), non SCs (S100-/RECA-), or macrophages ($Iba1^+$) to the closest blood vessel, Day 4 after transection (n = 4, graph shows mean value ± SEM).

(D) 3D-projection of a rat nerve bridge showing a S100-positive SC (green) interacting with a newly formed EdU-positive (red) blood vessel ($RECA\text{-}1^+$, white). Scale bar, 20 µm. See also Movie S1.

(E) Snapshots of a 3D-image obtained by the surface rendering of a z-stack projection of confocal images of the rat nerve bridge, immunostained to detect SCs ($S100^+$, green) and ECs ($RECA\text{-}1^+$, red). A SC can be seen to interact with two different blood vessels through cytoplasmic protrusions. Scale bar, 20 µm. Arrowheads indicate points of contact between a SC and blood vessels.

(F) Representative confocal image of a longitudinal section of a sciatic nerve bridge from PLP-EGFP mice, Day 5 after transection, immunostained to detect ECs ($CD31^+$, red). Scale bar, 50 µm.

(G) Snapshots from Movie S2 showing blebs and protrusions mediating the contacts between SCs and ECs within the bridge.

(H) Snapshots from Movie S1 of a 3D model obtained by surface rendering of a z stack projection of a longitudinal section of a bridge region from the sciatic nerve of PLP-EGFP mice, Day 5 after transection. The sections were immunostained to detect ECs ($CD\text{-}31^+$, blue) and axons (NF^+, red). Scale bar, 20 µm.
See also Figure S3.

of the cell rather than the forward extensions were inhibited by the addition of blebbistatin or the Rho-kinase inhibitor Y27632 demonstrating that actomyosin contractility is required for this mode of migration (Lämmermann et al., 2008) (Figure S4G; Movie S6). In contrast, latrunculin B also inhibited the forward protrusions showing actin structures were required for both cell movements (Figure S4G; Movie S6). Compared to cells migrating in 2D, focal adhesions were absent or extremely small in the 3D cultures (Figure S4H) and consistent with low levels of adhesion, whereas knockdown of beta1 integrin or talins severely inhibited SC migration in 2D, it had no effect on the ability of the same cells to migrate along the EC tubules (Figures S4I and S4J; Movie S7) (Bergert et al., 2015; Lämmermann et al., 2008).

Together with the EM analysis, these results indicate that while SCs are unable to generate sufficient force to migrate within the confinements of the 3D matrix, blood vessels provide a distinct confined environment and a sufficiently frictional or discontinuous surface that allows an actomyosin-driven, amoeboid-like mode of migration in the desired direction of travel (Bergert et al., 2015; Liu et al., 2015; Tozluoğlu et al., 2013).

Macrophages Are the Sensors of Hypoxia within the Bridge

New blood vessels normally form in response to decreased oxygen levels (hypoxia) within a tissue. Upon hypoxia, the transcription factor HIF-1α is stabilized and initiates a transcriptional response that induces angiogenesis by upregulating

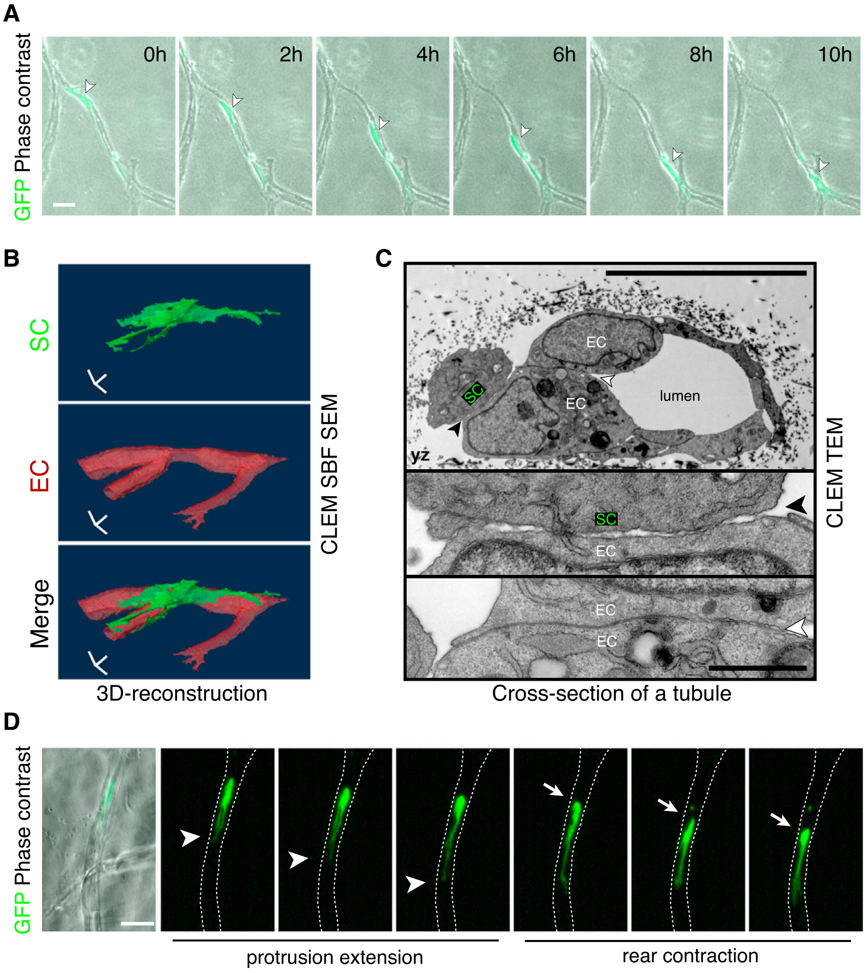

Figure 4. SCs Migrate along Endothelial Tubules In Vitro

(A) Representative time-lapse microscopy images showing a GFP-positive rat SC migrating along a tubule of HUVECs within a 3D fibrin gel (Movie S3). Scale bar, 40 μm. White arrowheads indicate the cell body of the SC.

(B) Images from Movie S4 of a tilted 3D view of a GFP-positive SC (green) interacting with an EC tubule (red). Scale bar, 10 μm.

(C) Top: a representative EM image of a cross-section of an EC tubule in contact with a GFP-positive SC within a fibrin gel. Scale bar, 10 μm. Middle: a higher magnification view of the SC/EC contact (black arrowhead). Bottom: a higher magnification view of the EC/EC contact (white arrowhead). Scale bar, 1 μm.

(D) Snapshots of Movie S5, showing the amoeboid-like mode of migration observed by the SCs in 3D. White arrowheads and arrows show the leading protrusion and the rear of the cell respectively. Scale bar, 50 μm.

See also Figure S4.

pro-angiogenic factors such as VEGF (Krock et al., 2011; Pugh and Ratcliffe, 2003). To test whether the nerve bridge was hypoxic, we injected rats with hypoxyprobe-1 (pimonidazole hydrochloride) that forms immunofluorescent detectable protein adducts in hypoxic conditions (pO_2 < 10 mm Hg) (Young and Möller, 2010). Immunostaining of day 2 nerve bridges revealed the presence of large numbers of hypoxic cells prior to its vascularization (Figures 5A and S5A). Hypoxic cells were found only in the bridge and at the tips of both the distal and proximal stumps but not further along the stumps or in the uncut nerve (Figure S5B). The proportion of hypoxic cells decreased substantially by day 3, when the bridge had become vascularized (Figures 5A and S5A), consistent with the new blood vessels resolving the hypoxic environment of this new tissue.

Remarkably, not all cells in the bridge were positive for the hypoxyprobe-1 at day 2, suggesting that certain cell types may be more sensitive to the hypoxia. As macrophages have been shown to promote angiogenesis during wound healing and within tumors (Murdoch et al., 2008; Rodero and Khosrotehrani, 2010) and comprise more than half of the cells in the bridge, we tested whether the hypoxic cells were macrophages. Co-labeling of hypoxic cells and macrophages showed that the vast majority (>98%) of hypoxic cells were macrophages (Figures 5B and S5C) and that most macrophages (~80%) were hypoxic on day 2 (Figure S5D). These observations indicate that macrophages are selectively sensing the hypoxic environment in the nerve bridge. To test whether this was an intrinsic property of the cells, we purified cells from the bridge and exposed them to varying oxygen concentrations in vitro. Consistent with our in vivo findings, we found that macrophages became hypoxic at higher oxygen concentrations (1.5%) than the other bridge cells (Figure 5C).

The hypoxia at day 2 was associated with increased HIF-1α levels in the bridge macrophages (Figure S5E). Moreover, in situ hybridization (Figure S5F), RT-qPCR (Figure S5G), and antibody staining (Figures S5H–S5L) demonstrated increased expression of VEGF-A in macrophages within the bridge consistent with a role for bridge-derived VEGF in stimulating EC proliferation and migration from the nerve stumps into the bridge.

While VEGF is a potent chemoattractant for ECs, we tested whether it also attracts SCs. Using a transwell assay, we found that ECs but not SCs migrated in response to VEGF-A, a response inhibited by the VEGFR2 inhibitor, cabozantinib (Figure 5D). We also found that conditioned medium from hypoxic purified bridge cells attracted ECs in a cabozantinib-dependent manner, whereas SC migration was independent of VEGF signaling but still responded to other factors secreted by the bridge cells or serum (Figure 5D). Together, these data suggest that after injury, ECs are specifically recruited from both nerve stumps in response to VEGF-A secretion by macrophages within the bridge. To test this in vivo, we treated mice with cabozantinib

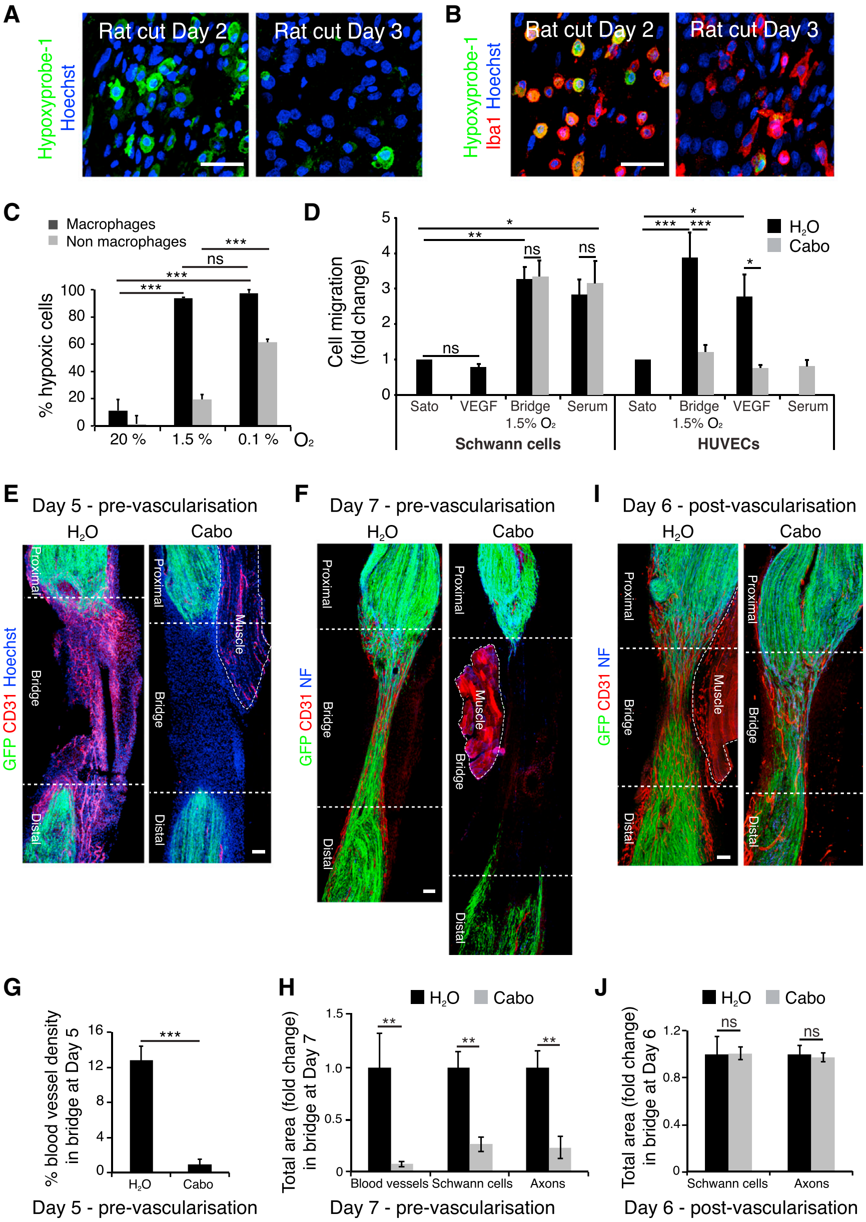

Figure 5. Hypoxia Drives Angiogenesis by a Macrophage-Generated Gradient of VEGF-A

(A) Representative images of sections of a rat sciatic nerve bridge, Day 2 and 3 after transection and 30 min after injection of hypoxyprobe-1, immunolabeled to detect hypoxyprobe-1 (green). Scale bar, 25 μm.

(B) As in (A) but immunolabeled to detect macrophages ($Iba1^+$, red) and hypoxic cells (hypoxyprobe-1^+, green). Scale bar, 25 μm.

(C) Graph showing percentage of hypoxic cells (hypoxyprobe-1^+) in macrophage ($Iba1^+$) and non-macrophage ($Iba1^-$) populations from rat sciatic nerve bridges cultured at indicated oxygen conditions (n = 3).

(D) HUVECs or SCs were placed in the upper compartment of Boyden chambers and allowed to migrate into the lower chamber containing media with no factors (SATO), VEGF-A^{165}, serum, or conditioned medium from bridge cells cultured at 1.5% O_2 (n = 5). For (C) and (D) one-way ANOVA test was used for statistical analysis.

(E–H) Confocal images of longitudinal cryosections of injured sciatic nerves from PLP-EGFP mice, Day 5 or Day 7 after transection, following gavage of cabozantinib or control solvent on Day 4 (pre-vascularization), immunostained to detect ECs ($CD31^+$, red) and axons (NF^+, blue) Scale bar, 50 μm, quantified in (G) and (H) (n = 3).

(I) As for (F) but cabozantinib was administered on Day 5 (post-vascularization) and harvested on Day 6, quantified in (J) (n = 3). For reconstruction of longitudinal sections shown in (E), (F), and (I), multiple images from the same sample were acquired using the same microscope settings.

Graphs show mean value ± SEM. See also Figure S5.

prior to blood vessel formation and found the inhibitor was able to block both blood vessel entry into the bridge and the subsequent entry of SCs and axons (Figures 5E–5H). Importantly however, the same inhibitor, added just after blood vessel formation, did not impair either SC or axonal entry (Figures 5I and 5J). These results indicate that VEGF-A is required for ECs to cross the bridge but that SCs and axons migrate independently of VEGF signaling once the blood vessels are formed.

Macrophages Drive Angiogenesis within the Bridge

To test the in vivo importance of macrophage-derived VEGF-A in promoting the vascularization of the bridge, we performed nerve transections in two distinct complementary mouse models in which *Vegfa* was inactivated in macrophages. We crossed floxed *Vegfa* conditional null mice (Gerber et al., 1999) with mice expressing CRE-recombinase under the control of the *Lysm* promoter to generate mice lacking *Vegfa* in most macrophages and granulocytes ($Vegfa^{fl/fl}$ $Lysm^{Cre}$) (Clausen et al., 1999) and to mice expressing CRE from the *Tie2* promoter to generate mice lacking *Vegfa* in hematopoietic cells and ECs ($Vegfa^{fl/fl}$ *Tie2-Cre*) (Fantin et al., 2010). The mice also contained a floxed YFP-reporter gene in the Rosa26 locus to monitor the efficiency of CRE-mediated recombination (Srinivas et al., 2001).

VEGF-A has been described to play a role in the recruitment of macrophages from the bloodstream (Cursiefen et al., 2004). We therefore tested whether their recruitment was inhibited within the bridge of the knockout animals. We quantified the number of macrophages within the bridges from control and mutant $Vegfa^{fl/fl}$ $Lysm^{Cre}$ and $Vegfa^{fl/fl}$ *Tie2-Cre* animals at day 5 and found no differences (Figure S6A) showing that loss of *Vegfa* does not impair macrophage recruitment during the early phase of nerve regeneration. We determined the efficiency of recombination and found that 82% of macrophages had been targeted

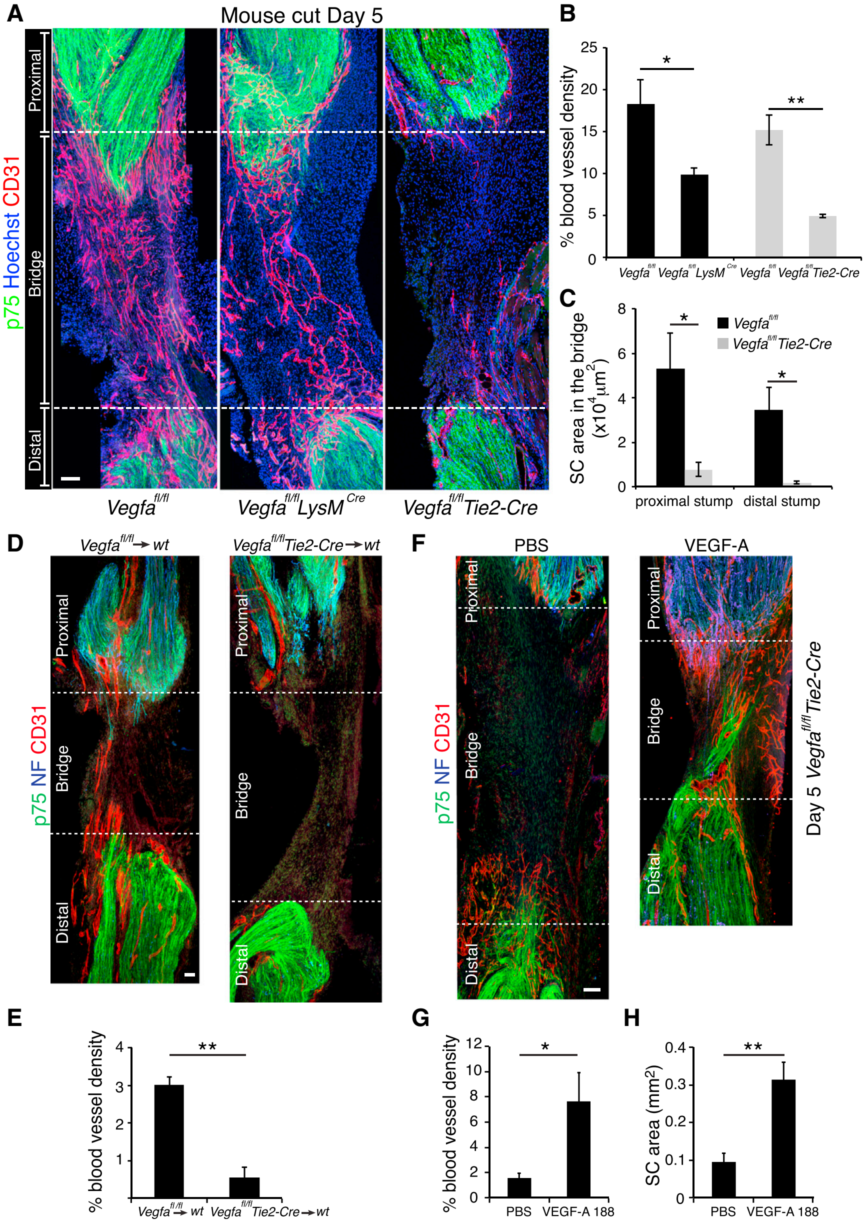

Figure 6. Inactivation of *Vegfa* in Macrophages Inhibits Vascularization of the Nerve Bridge after Nerve Transection

(A) Representative images of longitudinal sections of injured sciatic nerves from *Vegfa$^{fl/fl}$* (control), *Vegfa$^{fl/fl}$ Lysm^{Cre}*, and *Vegfa$^{fl/fl}$ Tie2-Cre* mice, Day 5 after transection, immunostained to detect ECs (CD31^{+}, red) and SCs (p75^{NTR+}, green). Scale bar, 50 μm.

(B) Quantification of (A) showing the proportion of CD31-positive area per bridge area and shows that the vascularization of the bridge is significantly reduced in mutants animals (n = 5).

(C) Quantification of (A) showing the area of SC influx from the proximal and distal stumps in *Vegfa$^{fl/fl}$* versus *Vegfa$^{fl/fl}$ Tie2-Cre* animals (n = 5).

(D) Representative images of longitudinal sections of injured sciatic nerves from wild-type that have received bone marrow from *Vegfa$^{fl/fl}$* (control) or *Vegfa$^{fl/fl}$ Tie2-Cre* mice immunostained to detect ECs (CD31^{+}, red), SCs (p75^{NTR+}, green), and axons (NF^{+}, blue), Day 5 after transection. Scale bar, 100 μm.

(E) Quantification of (D) showing the proportion of CD31-positive area per bridge area (n = 3 for each group).

(F) Representative images of longitudinal sections of injured sciatic nerves of *Vegfa$^{fl/fl}$ Tie2-Cre* mice, Day 5 after transection following injection of PBS or VEGF-A^{188} into the bridges at Day 4. Scale bar, 100 μm.

(G and H) Quantification of (F) showing the blood vessel density (G) or area of infiltrating SCs (H) (n = 4). For reconstruction of longitudinal sections shown in (A), (D) and (F), multiple images from the same sample were acquired using the same microscope settings.

Graphs show mean value ± SEM. See also Figure S6.

within the bridge of *Vegfa$^{fl/fl}$ Tie2-Cre* animals (Figure S6B). Consistent with this, we observed an ~80% decrease in *Vegfa* mRNA levels (Figure S6C). In contrast, the *Vegfa$^{fl/fl}$ Lysm^{Cre}* mutants showed a lower rate of recombination (42%) (Figure S6B).

Macrophages have been shown to promote angiogenesis (Fantin et al., 2010; Pollard, 2009) and autocrine VEGF-A signaling helps to maintain the health of ECs (Lee et al., 2007). We therefore analyzed the vasculature of uninjured nerves from all genotypes but found no differences (Figures S6D and S6E). Remarkably however, nerves from both mutant animals showed a reduction in the vascularization of the bridge following injury (Figure 6A). The extent of the inhibition was more dramatic in the *Vegfa$^{fl/fl}$ Tie2-Cre* mice, consistent with the greater degree of recombination in these animals, with very few blood vessels detectable within the bridge (Figure 6B). However, there was also a significant decrease in the *Vegfa$^{fl/fl}$ Lysm^{Cre}* mice (Figure 6B). Strikingly, SCs remained in the stumps of the *Tie2-Cre* mutant animals, consistent with a requirement for blood vessels to provide a "track" for the SCs to enter the bridge (Figure 6C). To confirm this was not due to loss of VEGF-A expression in ECs we (1) performed bone marrow transplant experiments from *Vegfa$^{fl/fl}$ Tie2-Cre* and control *Vegfa$^{fl/fl}$* litter-mates into WT mice and found similar defective entry of blood vessels into the bridges of the mice receiving the mutant bone marrow, confirming that cells derived from hematopoietic-stem cells were responsible for the defect (Figures 6D, 6E, S6F, and S6G); and (2) performed rescue experiments in the *Vegfa$^{fl/fl}$ Tie2-Cre* mice. We injected either VEGF-A or PBS into the bridges of *Vegfa$^{fl/fl}$ Tie2-Cre* mice on day 4 and found that VEGF-A was able to rescue EC migration into the bridge and that SCs and axons migrated along these blood vessels (Figures 6F–6H). These results show that ECs deleted for VEGF-A are able to migrate and survive in the bridge and also

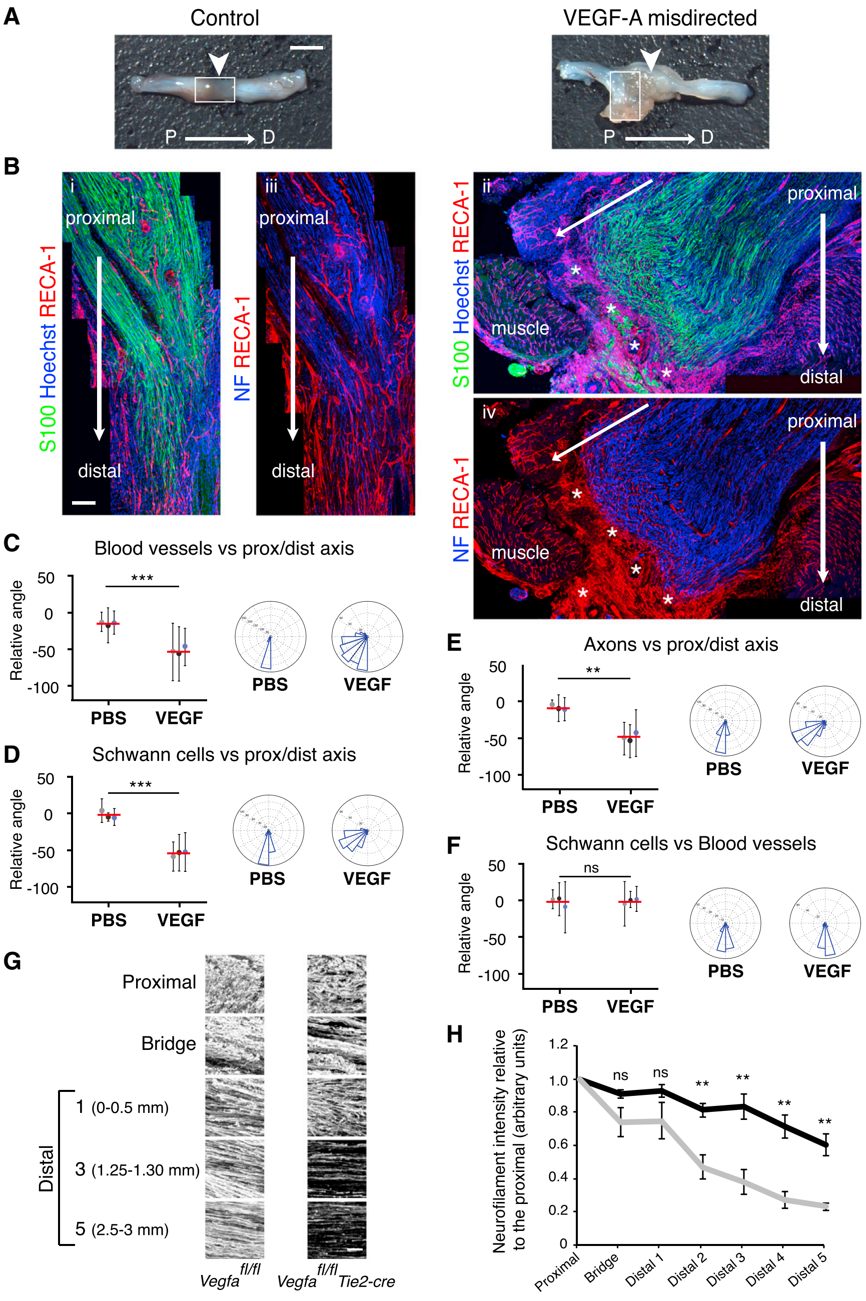

Figure 7. Redirection of the Blood Vessels Leads to the Misdirection of Migrating SCs

(A) PBS- (control) and VEGF-treated rat sciatic nerve images show that placement of VEGF beads to the side of the injury site, leads to aberrant regeneration. Scale bar, 2 mm. Arrows indicate the bridge region and proximal to distal.

(B) Immunofluorescence images of the regions demarcated by white boxes in (A) of a PBS- (control) and VEGF-treated animal, Day 6 following injury, longitudinal sections were immunostained to detect SCs ($S100^{+}$, green) and ECs ($RECA1^{+}$, red). i and ii: show that misdirected blood vessels in the VEGF-treated animals directed the SC cords toward the adjacent muscle. iii and iv: show the axons (NF^{+}) following the SC cords, toward the muscle. Scale bar, 300 μm. White asterisks indicate the beads. For reconstruction of longitudinal sections, multiple images from the same sample were acquired using the same microscope settings.

(C–F) Quantification of (B) to show the direction of blood vessels (C), SCs (D), and axons (E) relative to the proximal/distal axis and the alignment of blood vessels and SCs (F) in the rats treated with PBS or VEGF (n = 3). Graphs show the mean relative angle ± SD for each animal with the mean between animals shown by red lines. Rose plots show the distribution of cells for all animals.

(G) Representative confocal images of axons (NF^{+}) in indicated regions of regenerated nerves in *Vegfa*$^{fl/fl}$ (control) or *Vegfa*$^{fl/fl}$ *Tie2-Cre* mice, Day 14 after transection.

(H) Quantification of (G) showing axonal growth in *Vegfa*$^{fl/fl}$ (black line) and *Vegfa*$^{fl/fl}$ Tie2-cre (gray line) mice (n = 5, graph shows mean value ± SEM).

See also Figure S7.

provide a substrate for SC migration. Together, these results show that macrophages in the bridge secrete VEGF-A to enable the formation of a polarized endothelial scaffold that can direct SCs out of the nerve stumps and across the bridge.

Schwann Cells Use the Polarized Vasculature as a Scaffold to Guide Regrowing Axons

To address whether VEGF-A-induced blood vessels are sufficient to guide cords of SCs, we redirected the blood vessels to test whether the SCs would follow the blood vessels or continue to cross the bridge. To do this, we implanted heparin beads loaded with recombinant human $VEGF^{165}$ into muscle adjacent to the proximal side of the injury site, immediately after the transection of the rat sciatic nerve. Six days later, the regenerative process was found to be abnormal in 10 out of 13 of the VEGF-treated animals compared to 1 out of 13 PBS-bead-treated controls. In five of the ten VEGF-treated animals in which abnormal regeneration was observed, a complete failure of the regenerative process was associated with misdirection of the blood vessels, SC cords and the accompanying axons, away from the bridge and into surrounding muscle towards the beads (Figures 7A, 7B, and S7A; quantified in Figures 7C–7F and S7B). Analysis of the bridges in a further five cases showed that the beads had moved into the bridge leading to the formation of disorganized blood vessels close to the beads (Figure S7C). In these cases, SCs migrated into the vascularized areas and either appeared "trapped" or deviated from the normal direction of movement, taking the axons along with them (Figure S7D). Moreover, beads implanted adjacent to the distal stump could also redirect blood vessels and SCs

(Figure S7E). Together, these results demonstrate that VEGF-induced blood vessels are sufficient to guide SCs and their accompanying axons during peripheral nerve regeneration.

Finally, to address whether disruption of this process leads to long-term defects in the regeneration of a peripheral nerve, we analyzed the nerves of the *Vegfa*$^{fl/fl}$ *Tie2-Cre* mice at later points following injury. At day 14, while some axons succeeded in crossing the bridge in the mutant mice, a lower number of axons regrew into the distal stump compared to controls (Figures 7G and 7H). This difference is reflected at a later time-point (6 months), when the regenerated distal regions of mutant nerves were visibly smaller (Figures S7F–S7H). As the structure of the nerve within the regenerated regions was indistinguishable from controls (Figure S7G), this most likely reflects a remodeling of the nerve in response to the lower number of axons crossing the bridge and entering into the distal stump. This phenotype shows that the efficient construction of the endothelial bridge structure is essential for the effective regeneration of peripheral nerve.

DISCUSSION

The regeneration of a tissue following an injury requires the repair or replacement of the damaged or lost cellular structures. In some animals, such as newts and salamanders, this process is highly efficient, in that limbs and other organs can be regenerated following their loss after injury. This involves reprogramming of cells at the injury site back to a multipotent progenitor state with encoded positional information that allows the recapitulation of developmental processes to regenerate the lost cellular structures (Poss, 2010). In adult mammals, for reasons that remain unclear, regeneration is limited and seems mostly to take place within the confines of existing tissue structures and involves mechanisms distinct from those used during development. Peripheral nerves, unlike those of the CNS, are one of the few tissues in mammals capable of extensive regeneration, in that even following an injury as severe as a complete transection, the damaged nerves are able to reconnect with their original targets (Nguyen et al., 2002). However, this does not involve regeneration of the entire nerve structure downstream of the cut instead the distal stump remains intact, reconnects with the proximal stump via a "bridge" of new tissue, and is remodeled to provide a suitable environment to guide and support regrowing axons back to their targets. A major hurdle in this process is the guidance and support of the axons across the "bridge" prior to entering into the distal stump of the nerve and it has been unclear how this is achieved.

In rodents, reconnection of the two nerve stumps occurs naturally but involves the formation of a relatively long "bridge" of new tissue, a seemingly hostile environment consisting of inflammatory cells and matrix across which the axons need to find their way, in the absence of the guidance cues that were present during development. In a previous study, we showed that at the wound site, SCs were marshalled into cords following interactions with fibroblasts as the result of ephrinB/EphB2 signaling between the two cell types (Parrinello et al., 2010). The cords of SCs were found to be critical for transporting the axons across the bridge but left open the question of how the cords of SCs manage to find their way. In this study, we have identified two additional processes that are required to direct the SCs. The first involves macrophages within the bridge responding to the hypoxic environment by secreting VEGF-A and thereby stimulating the formation of blood vessels that orientate in the direction of subsequent travel. The second involves the SC cords using the polarized blood vessels as a migratory scaffold to enter and cross the bridge.

Interestingly, while the bridge contains multiple cell types, only macrophages exhibited a detectable hypoxic response, showing that distinct cell types respond differentially to a hypoxic environment and indicating a specific role for macrophages in directing the regeneration process. The macrophages display a classical hypoxic response involving the stabilization of HIF-1α and increased VEGF-A levels, and it remains unclear why only macrophages respond despite being in the same environment as the other cell types. Yet the response appears to be an intrinsic property of the cells as we found it can be reproduced in vitro. The critical role of macrophages in triggering the angiogenic response was confirmed by our findings that loss of *Vegfa* from myeloid cells blocked both the angiogenic response and subsequent SC entry into the bridge. Other studies have also reported a role for macrophages in inducing blood vessels following injury, suggesting that this may be a general mechanism (Wynn et al., 2013). For example, macrophages promote angiogenesis following a wound to the skin, although it was not reported whether the macrophages responded differentially to the hypoxic environment than other cell types in the wound (Rodero and Khosrotehrani, 2010). Moreover, during tumor development, hypoxia leads to the accumulation of macrophages that, via VEGF secretion, promote the vascularization of tumors (Murdoch et al., 2008; Qian and Pollard, 2010). Macrophages may therefore play a general role as primary sensors of hypoxia to induce neovascularization in the adult. It would be of great interest to characterize the mechanisms by which macrophages specifically sense the hypoxic environment and to determine the generality of this phenomenon following injury and in pathological conditions.

The newly formed blood vessels within the bridge provide a scaffold for the migrating SC cords. The importance of this appears to be twofold. First, SCs appear unable to migrate within the 3D matrix but instead require the physical surface of blood vessels in order to migrate efficiently. The amoeboid-like mode of migration observed by our live-imaging is seen in conditions of low adhesion and higher levels of confinement (Lämmermann and Sixt, 2009; Liu et al., 2015) and consistent with this, we find migration appears independent of the focal adhesions required for migration of SCs in 2D while dependent on actomyosin contractility from the rear of the cell. Moreover, recent studies describe that non-specific blebs coupled to a discontinuous environment are sufficient to provide propelling forces for migration in 3D (Bergert et al., 2015; Liu et al., 2015; Tozluoğlu et al., 2013), and we observe multiple blebs providing points of contact between the migrating SCs and ECs. Together, these findings indicate that SCs, unlike fibroblasts and ECs, cannot generate sufficient force to migrate through a 3D matrix and, while there may be specific molecular interactions between the SCs and ECs, our results are

consistent with the blood vessels providing non-specific friction and a surrounding environment that allows actomyosin-driven migration along their surface.

Second, the blood vessels provide directionality to the SC movement—this is clearly demonstrated by our findings that redirection of the blood vessels can lead SC cords out of the nerve and into surrounding tissue. The requirement of a cellular substrate for cells to migrate in vivo may be a more general phenomenon than is broadly appreciated. During development, many cell types travel substantial distances and many appear to use pre-existing cell structures as a substrate to find their way. For example, during development, lymphatic ECs have been shown to migrate along pre-existing arteries (Bussmann et al., 2010) and neuroblasts migrate along radial glia (Nadarajah and Parnavelas, 2002). In the adult, mass migrations are less frequent but are associated with pathological conditions. For example during adult neurogenesis neuroblasts migrate along blood vessels (Bovetti et al., 2007), a process enhanced after a stroke (Kojima et al., 2010). In addition, there are increasing reports that tumor cells use the vasculature as a means to migrate away from the site of the primary tumor. Glioma cells are frequently highly invasive and appear to migrate along pre-existing vasculature to spread within the brain (Farin et al., 2006). Moreover, melanoma cells have also been seen to disseminate using the vasculature as a substrate (Lugassy and Barnhill, 2007) and metastatic cells appear to establish lesions in the brain along the vasculature (Carbonell et al., 2009). Identification of the mechanisms responsible for these processes is therefore likely to be of therapeutic interest.

While many nerve injuries in humans can be aided by surgery, a major therapeutic problem is the frequent loss of segments of nerve at the site of injury (Pfister et al., 2007). To overcome this problem, attempts are made to bridge the gap by the use of nerve grafts or artificial nerve conduits, however, the efficiency of axonal regrowth across the injury site is often poor. Our results suggest that encouraging or mimicking a polarized vasculature within the grafts (Hobson et al., 1997), could increase the efficiency of this process by encouraging SC entry into the bridge to provide a more conducive environment for axonal regrowth.

EXPERIMENTAL PROCEDURES

Animals

Animal work was carried out in accordance to regulations of the UK Home Office. Adult (6- to 8-week-old) Sprague-Dawley male rats and 4- to 6-week-old mice were used for all experiments. To delete *Vegfa* in macrophages, we crossed floxed Vegfa (*Vegfafl/fl)* mice (Gerber et al., 1999) with mice carrying the *Tie2-Cre* transgene (Kisanuki et al., 2001) or the knock-in $Lysm^{Cre}$ (Clausen et al., 1999). These mice also contained the floxed *Rosa26Yfp* reporter (Srinivas et al., 2001). PLP-EGFP transgenic mice (Mallon et al., 2002) were used for studies requiring GFP^+ SCs.

In Vivo Analysis

Sciatic nerves were exposed under general anesthesia in aseptic conditions and transected at mid-thigh. Nerves were dissected at the indicated days for analysis by immunostaining or EM. For immunostaining, pre- or post-fixed longitudinal sections of the sciatic nerves were immunostained as detailed in the Supplemental Experimental Procedures and analyzed using confocal microscopy. Blood vessel density and the area occupied by SCs and axons within the nerve bridge, was quantified using Fiji. For analysis of cell proliferation, EdU incorporation was measured using the Click-iT cell proliferation assay kit (Invitrogen). For analysis of hypoxia, the hypoxyprobe-1 kit (hypoxyprobe) was used according to the manufacturer's instructions. To determine the functionality of the blood vessels, fluorescein-conjugated *Griffonia* Simplicifolia lectin I (Vector Lab) was injected into the tail vein prior to harvesting. For EM analysis, fixed nerves were embedded in Epon and analyzed for TEM or CLEM as detailed in the Supplemental Experimental Procedures. 3D reconstruction of confocal images of the nerve bridge in both rats and mice was performed using Fiji and Imaris software. 3D reconstruction of TEM images was performed using Amira software.

Bone Marrow Transplantation

Bone marrow cells (3×10^6) from donor mice ($Vegfa^{fl/fl}$ control and $Vegfa^{fl/fl}$ *Tie2-Cre* mutant) were injected into the tail vein of lethally irradiated recipient WT mice. To check the engraftment efficiency, peripheral blood was collected after 4 weeks and analyzed by FACS. Only mice with higher than 90% engraftment of donor cells were used.

In Vivo Rescue, Inhibitor, and Bead Studies

To inhibit VEGFR, PLP-EGFP mice were orally administrated with indicated doses of 100 mg/kg of Cabozantinib and harvested at the indicated times. To rescue the loss of *Vegfa*, nerves bridges of $Vegfa^{fl/fl}$ *Tie2-Cre* mice day 4 after injury were re-exposed under general anesthesia and injected with 5 μl of VEGF-A^{188} or PBS 1 day before harvesting. To redirect the blood vessels, $VEGF^{165}$- or PBS-coated heparin beads were placed to the side of the injury site within adjacent muscle.

Cells

Rat SCs and fibroblasts were cultured from P7 animals as described (Mathon et al., 2001). HUVECs were cultured in endothelial cell growth medium-2 (ECGM-2, PromoCell). Immortalized human dermal fibroblasts (HDFs) were cultured as primary rat fibroblasts.

In Vitro Migration Assays

The fibrin gel bead assay was performed as described (Nakatsu et al., 2003). GFP-expressing SCs were added to the beads prior to thrombin addition at a final concentration of 1.25×10^4 cells/ml. Time-lapse microscopy or immunofluorescence analysis was performed 10 days later with or without the presence of indicated inhibitors. For Matrigel assays, 150 μl of Matrigel (BD) was added to a 24-well plate and 2.5×10^4 HUVECs were seeded. Fourteen hours later, 5×10^3 GFP SCs were added to the Matrigel and time-lapse microscopy was performed.

In Vitro Bridge Analysis

Rat nerve bridges at day 2 following injury were collected and enzymatically digested. For hypoxia analysis, 6×10^4 cells were seeded in a 24-well plate and incubated overnight. The cells were then incubated at 20%, 1.5%, or 0.1% O_2 for 4 hr in the presence of 100 μM pimonidazole HCl. For cell migration analysis, 10^5 nerve bridge cells were incubated in minimal media in the bottom of transwells for 24 hr. SCs and HUVECs were then added to the fibronectin-coated inserts and allowed to migrate for 4 hr at 20% O_2.

Statistical Analysis

All data are represented as mean values ± SEM unless indicated otherwise. Unpaired two-tailed Student's t tests were used for statistical analysis unless indicated otherwise and p values are indicated by asterisks as follows: *$p < 0.05$, **$p < 0.01$, ***$p < 0.001$.

SUPPLEMENTAL INFORMATION

Supplemental Information includes Supplemental Experimental Procedures, seven figures, and seven movies and can be found with this article online at http://dx.doi.org/10.1016/j.cell.2015.07.021.

ACKNOWLEDGMENTS

This work was supported by a programme grant from CRUK (C378/A17135). I.N. was supported by Worldwide Cancer Research. F.M. and C.R. were supported by the Biotechnology and Biological Sciences Research Council (BBSRC) (BB/J00930X/1) and the Rosetrees Trust (A533). We thank UCL Biological Services for the maintenance of the animals and useful advice, W. Macklin and M. Kerschensteiner for the PLP-EGFP mice, N. Ferrara and Genentech for the *Vegfa* floxed mice, K. Davidson for help with genotyping, A. Fantin for helpful suggestions, S. Mole for HDFs, T. Nightingale for HUVECs, P. Munro for assistance with SBF/SEM, and E. Paluch, Y. Mao, and M. Tozluoğlu for advice on cell migration. A.C.L. would like to dedicate this work to the memory of Alan Hall and Chris Marshall, who both mentored her throughout her career.

Received: April 19, 2015
Revised: June 11, 2015
Accepted: June 30, 2015
Published: August 13, 2015

REFERENCES

Avellino, A.M., Hart, D., Dailey, A.T., MacKinnon, M., Ellegala, D., and Kliot, M. (1995). Differential macrophage responses in the peripheral and central nervous system during wallerian degeneration of axons. Exp. Neurol. *136*, 183–198.

Bergert, M., Erzberger, A., Desai, R.A., Aspalter, I.M., Oates, A.C., Charras, G., Salbreux, G., and Paluch, E.K. (2015). Force transmission during adhesion-independent migration. Nat. Cell Biol. *17*, 524–529.

Bovetti, S., Hsieh, Y.C., Bovolin, P., Perroteau, I., Kazunori, T., and Puche, A.C. (2007). Blood vessels form a scaffold for neuroblast migration in the adult olfactory bulb. J. Neurosci. *27*, 5976–5980.

Bryant, D.M., and Mostov, K.E. (2008). From cells to organs: building polarized tissue. Nat. Rev. Mol. Cell Biol. *9*, 887–901.

Bussmann, J., Bos, F.L., Urasaki, A., Kawakami, K., Duckers, H.J., and Schulte-Merker, S. (2010). Arteries provide essential guidance cues for lymphatic endothelial cells in the zebrafish trunk. Development *137*, 2653–2657.

Carbonell, W.S., Ansorge, O., Sibson, N., and Muschel, R. (2009). The vascular basement membrane as “soil” in brain metastasis. PLoS ONE *4*, e5857.

Clausen, B.E., Burkhardt, C., Reith, W., Renkawitz, R., and Förster, I. (1999). Conditional gene targeting in macrophages and granulocytes using LysMcre mice. Transgenic Res. *8*, 265–277.

Cursiefen, C., Chen, L., Borges, L.P., Jackson, D., Cao, J., Radziejewski, C., D’Amore, P.A., Dana, M.R., Wiegand, S.J., and Streilein, J.W. (2004). VEGF-A stimulates lymphangiogenesis and hemangiogenesis in inflammatory neovascularization via macrophage recruitment. J. Clin. Invest. *113*, 1040–1050.

Dudanova, I., and Klein, R. (2013). Integration of guidance cues: parallel signaling and crosstalk. Trends Neurosci. *36*, 295–304.

Fantin, A., Vieira, J.M., Gestri, G., Denti, L., Schwarz, Q., Prykhozhij, S., Peri, F., Wilson, S.W., and Ruhrberg, C. (2010). Tissue macrophages act as cellular chaperones for vascular anastomosis downstream of VEGF-mediated endothelial tip cell induction. Blood *116*, 829–840.

Farin, A., Suzuki, S.O., Weiker, M., Goldman, J.E., Bruce, J.N., and Canoll, P. (2006). Transplanted glioma cells migrate and proliferate on host brain vasculature: a dynamic analysis. Glia *53*, 799–808.

Fawcett, J.W., and Keynes, R.J. (1990). Peripheral nerve regeneration. Annu. Rev. Neurosci. *13*, 43–60.

Gerber, H.P., Hillan, K.J., Ryan, A.M., Kowalski, J., Keller, G.A., Rangell, L., Wright, B.D., Radtke, F., Aguet, M., and Ferrara, N. (1999). VEGF is required for growth and survival in neonatal mice. Development *126*, 1149–1159.

Hakkinen, K.M., Harunaga, J.S., Doyle, A.D., and Yamada, K.M. (2011). Direct comparisons of the morphology, migration, cell adhesions, and actin cytoskeleton of fibroblasts in four different three-dimensional extracellular matrices. Tissue Eng. Part A *17*, 713–724.

Heermann, S., and Schwab, M.H. (2013). Molecular control of Schwann cell migration along peripheral axons: keep moving!. Cell Adhes. Migr. *7*, 18–22.

Hobson, M.I., Brown, R., Green, C.J., and Terenghi, G. (1997). Inter-relationships between angiogenesis and nerve regeneration: a histochemical study. Br. J. Plast. Surg. *50*, 125–131.

Jurecka, W., Ammerer, H.P., and Lassmann, H. (1975). Regeneration of a transected peripheral nerve. An autoradiographic and electron microscopic study. Acta Neuropathol. *32*, 299–312.

Kisanuki, Y.Y., Hammer, R.E., Miyazaki, J., Williams, S.C., Richardson, J.A., and Yanagisawa, M. (2001). Tie2-Cre transgenic mice: a new model for endothelial cell-lineage analysis in vivo. Dev. Biol. *230*, 230–242.

Kojima, T., Hirota, Y., Ema, M., Takahashi, S., Miyoshi, I., Okano, H., and Sawamoto, K. (2010). Subventricular zone-derived neural progenitor cells migrate along a blood vessel scaffold toward the post-stroke striatum. Stem Cells *28*, 545–554.

Krock, B.L., Skuli, N., and Simon, M.C. (2011). Hypoxia-induced angiogenesis: good and evil. Genes Cancer *2*, 1117–1133.

Lämmermann, T., and Sixt, M. (2009). Mechanical modes of ‘amoeboid’ cell migration. Curr. Opin. Cell Biol. *21*, 636–644.

Lämmermann, T., Bader, B.L., Monkley, S.J., Worbs, T., Wedlich-Söldner, R., Hirsch, K., Keller, M., Förster, R., Critchley, D.R., Fässler, R., and Sixt, M. (2008). Rapid leukocyte migration by integrin-independent flowing and squeezing. Nature *453*, 51–55.

Lee, S., Chen, T.T., Barber, C.L., Jordan, M.C., Murdock, J., Desai, S., Ferrara, N., Nagy, A., Roos, K.P., and Iruela-Arispe, M.L. (2007). Autocrine VEGF signaling is required for vascular homeostasis. Cell *130*, 691–703.

Liu, Y.J., Le Berre, M., Lautenschlaeger, F., Maiuri, P., Callan-Jones, A., Heuzé, M., Takaki, T., Voituriez, R., and Piel, M. (2015). Confinement and low adhesion induce fast amoeboid migration of slow mesenchymal cells. Cell *160*, 659–672.

Lugassy, C., and Barnhill, R.L. (2007). Angiotropic melanoma and extravascular migratory metastasis: a review. Adv. Anat. Pathol. *14*, 195–201.

Mallon, B.S., Shick, H.E., Kidd, G.J., and Macklin, W.B. (2002). Proteolipid promoter activity distinguishes two populations of NG2-positive cells throughout neonatal cortical development. J. Neurosci. *22*, 876–885.

Martin, P., and Parkhurst, S.M. (2004). Parallels between tissue repair and embryo morphogenesis. Development *131*, 3021–3034.

Mathon, N.F., Malcolm, D.S., Harrisingh, M.C., Cheng, L., and Lloyd, A.C. (2001). Lack of replicative senescence in normal rodent glia. Science *291*, 872–875.

Murdoch, C., Muthana, M., Coffelt, S.B., and Lewis, C.E. (2008). The role of myeloid cells in the promotion of tumour angiogenesis. Nat. Rev. Cancer *8*, 618–631.

Nadarajah, B., and Parnavelas, J.G. (2002). Modes of neuronal migration in the developing cerebral cortex. Nat. Rev. Neurosci. *3*, 423–432.

Nakatsu, M.N., Sainson, R.C., Aoto, J.N., Taylor, K.L., Aitkenhead, M., Pérez-del-Pulgar, S., Carpenter, P.M., and Hughes, C.C. (2003). Angiogenic sprouting and capillary lumen formation modeled by human umbilical vein endothelial cells (HUVEC) in fibrin gels: the role of fibroblasts and Angiopoietin-1. Microvasc. Res. *66*, 102–112.

Napoli, I., Noon, L.A., Ribeiro, S., Kerai, A.P., Parrinello, S., Rosenberg, L.H., Collins, M.J., Harrisingh, M.C., White, I.J., Woodhoo, A., and Lloyd, A.C. (2012). A central role for the ERK-signaling pathway in controlling Schwann cell plasticity and peripheral nerve regeneration in vivo. Neuron *73*, 729–742.

Nguyen, Q.T., Sanes, J.R., and Lichtman, J.W. (2002). Pre-existing pathways promote precise projection patterns. Nat. Neurosci. *5*, 861–867.

Parrinello, S., Napoli, I., Ribeiro, S., Wingfield Digby, P., Fedorova, M., Parkinson, D.B., Doddrell, R.D., Nakayama, M., Adams, R.H., and Lloyd, A.C. (2010). EphB signaling directs peripheral nerve regeneration through Sox2-dependent Schwann cell sorting. Cell *143*, 145–155.

Pfister, L.A., Papaloïzos, M., Merkle, H.P., and Gander, B. (2007). Nerve conduits and growth factor delivery in peripheral nerve repair. J. Peripher. Nerv. Syst. *12*, 65–82.

Pollard, J.W. (2009). Trophic macrophages in development and disease. Nat. Rev. Immunol. *9*, 259–270.

Poss, K.D. (2010). Advances in understanding tissue regenerative capacity and mechanisms in animals. Nat. Rev. Genet. *11*, 710–722.

Pugh, C.W., and Ratcliffe, P.J. (2003). Regulation of angiogenesis by hypoxia: role of the HIF system. Nat. Med. *9*, 677–684.

Qian, B.Z., and Pollard, J.W. (2010). Macrophage diversity enhances tumor progression and metastasis. Cell *141*, 39–51.

Rodero, M.P., and Khosrotehrani, K. (2010). Skin wound healing modulation by macrophages. Int. J. Clin. Exp. Pathol. *3*, 643–653.

Srinivas, S., Watanabe, T., Lin, C.S., William, C.M., Tanabe, Y., Jessell, T.M., and Costantini, F. (2001). Cre reporter strains produced by targeted insertion of EYFP and ECFP into the ROSA26 locus. BMC Dev. Biol. *1*, 4.

Tozluoǧlu, M., Tournier, A.L., Jenkins, R.P., Hooper, S., Bates, P.A., and Sahai, E. (2013). Matrix geometry determines optimal cancer cell migration strategy and modulates response to interventions. Nat. Cell Biol. *15*, 751–762.

Wynn, T.A., Chawla, A., and Pollard, J.W. (2013). Macrophage biology in development, homeostasis and disease. Nature *496*, 445–455.

Young, R.J., and Möller, A. (2010). Immunohistochemical detection of tumour hypoxia. Methods Mol. Biol. *611*, 151–159.

Zochodne D.W., ed. (2008). Neurobiology of Peripheral Nerve Regeneration, First Edition (Cambridge University Press).

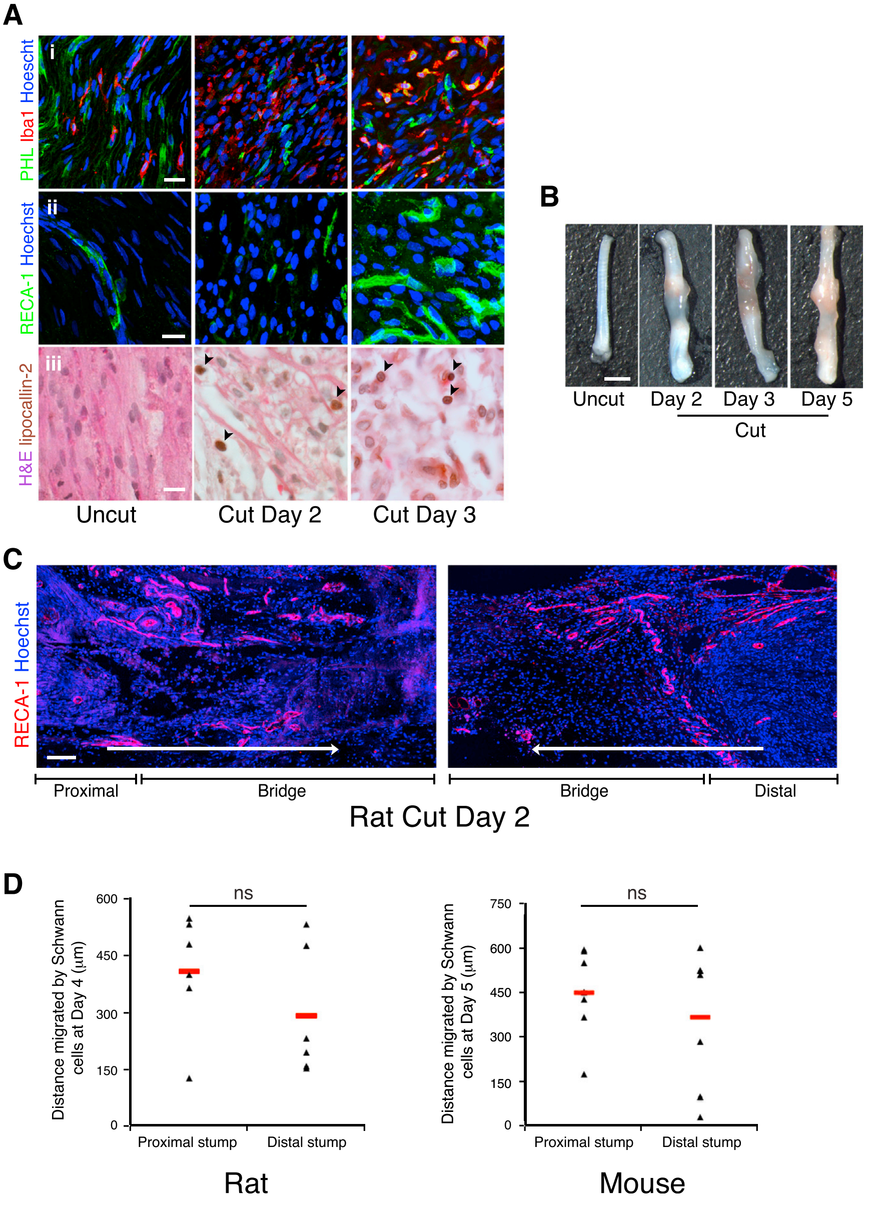

Figure S1. The Major Cellular Components of the Bridge Are Macrophages, Neutrophils, Fibroblasts, and Endothelial Cells, Related to Figure 1

(A) Representative images of longitudinal sections of rat uninjured and injured sciatic nerve bridges at Day 2 and 3. Sections were immunostained for (i) macrophages (Iba1+, red) and fibroblasts (prolylhydroxylase (PHL)+/ Iba1-, green). Scale bar = 25 μm. (ii) endothelial cells (RECA-1+, green). Scale bar = 10 μm. Nuclei were counterstained with Hoechst (blue). (iii) neutrophils - immunohistochemistry to detect lipocallin-2 (brown). Nuclei and cytoplasm were counterstained with Hematoxylin (violet) and Eosin (pink) respectively. Scale bar = 10 μm. Black arrowheads indicate lipocallin-2+ neutrophils. Quantification of the proportion of each cell-type within the bridge is shown in Figure 1A.

(B) Representative images of uninjured and injured rat sciatic nerves at Day 2, Day 3 and Day 5. Blood vessels can be observed within the bridge at Day 3 but not at Day 2.

(C) Representative immunofluorescence images of longitudinal sections of injured rat sciatic nerve bridges at Day 2 following transection, immunostained to detect blood vessels (RECA-1+, red) with nuclei stained with Hoechst. The images show the blood vessels entering the bridge from both the proximal stump (P) (left panel) and the distal stump (D) (right panel). Scale bar = 100 μm. Arrows indicates the direction of cell movement from the proximal or the distal stump into the bridge. For reconstruction of longitudinal sections, multiple images from the same sample were acquired using the same microscope settings.

(D) Schwann cells migrate from both the proximal and distal stumps. Graphs show the distance migrated by Schwann cells from the proximal and distal stumps in rats (LHS) and mice (RHS). Each point represents an individual nerve; red lines indicate the mean.

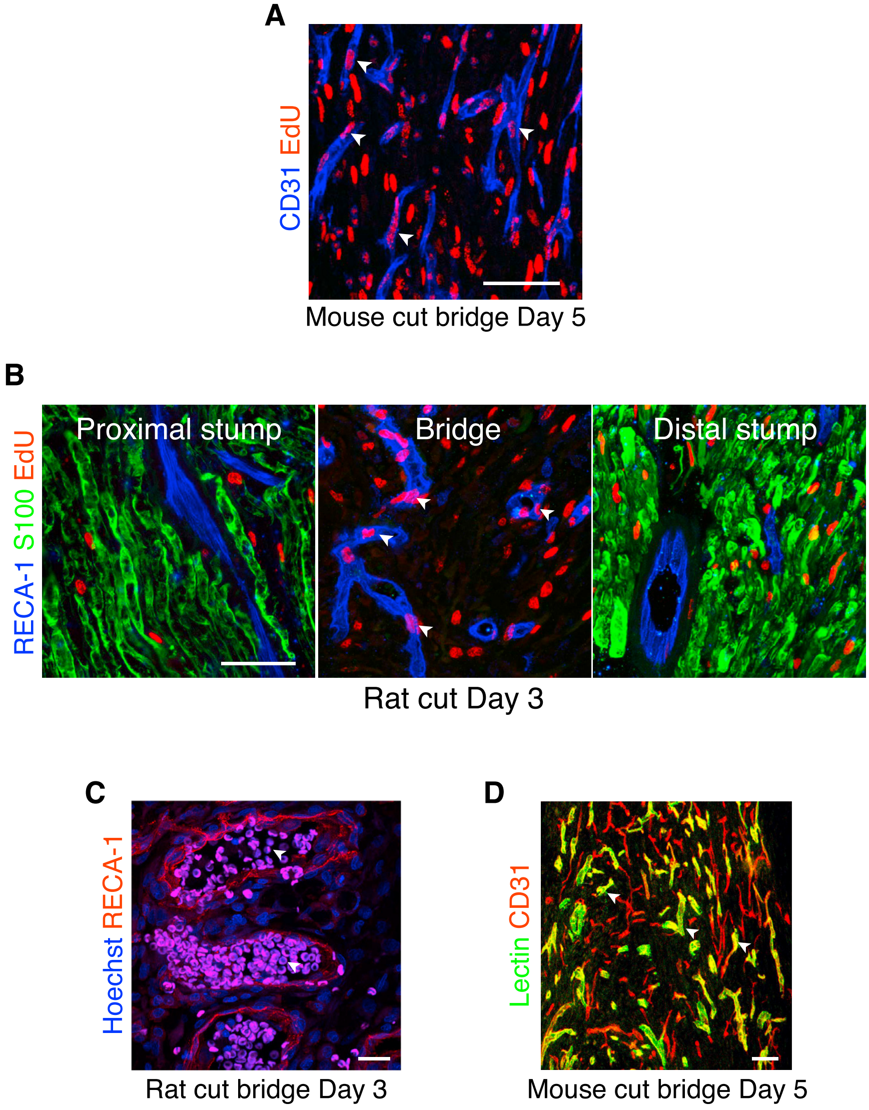

Figure S2. The Newly Formed Blood Vessels in the Bridge Are Functional, Related to Figure 2

(A) Representative image of a longitudinal section of a mouse sciatic nerve bridge, Day 5 after transection and 12 hr after EdU injection, immunostained to detect EdU (red), endothelial cells (CD31+, blue) to identify the presence of newly-formed blood vessels. Scale bar = 50 μm. White arrowheads indicate EdU+ endothelial cells.

(B) Representative longitudinal cryosections of the nerve stumps and the bridge of rat sciatic nerves, 12 hr after EdU injection and Day 3 after transection, immunostained for endothelial cells (RECA-1+, blue), Schwann cells (S100+, green) and EdU (red). Scale bar = 50 μm. White arrowheads indicate EdU+ endothelial cells.

(C) Representative immunofluorescence image of the bridge of injured rat sciatic nerves at Day 3 showing blood vessels immunostained for RECA-1 and autofluorescent erythrocytes, present in the vast majority of blood vessels. Scale bar = 25 μm. White arrowheads indicate erythrocytes within the lumen of the bridge vasculature.

(D) Representative longitudinal section of the bridge of an injured mouse sciatic nerve at Day 5 and 10 min after lectin-FITC injection and co-labeled to detect endothelial cells (CD31+, red). Scale bar = 50 μm. White arrowheads indicate functional blood vessels.

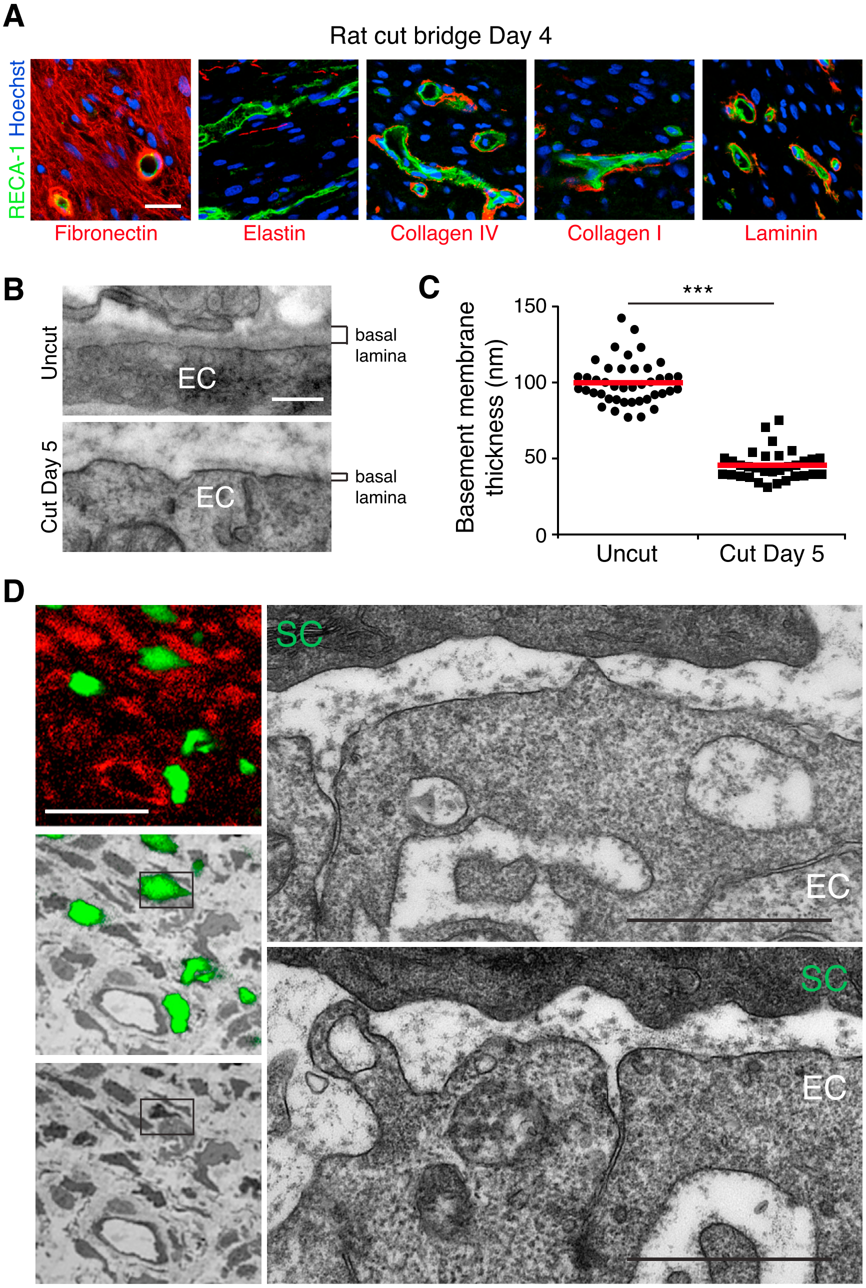

Figure S3. Blood Vessels in the Bridge Have Thin Basal Lamina, Allowing Direct Points of Contact with Schwann Cells, Related to Figure 3

(A) Representative confocal images of the bridges of rat sciatic nerves, Day 4 after transection, immunostained to detect the indicated matrix proteins (red) and endothelial cells (green). Scale bar = 25 μm.

(B) Representative TEM images of blood vessels from the bridge region and the contralateral nerve, Day 5 after transection. Note the basal lamina is thinner, less dense and/or absent around blood vessels within the bridge. Scale bar = 250nm.

(C) Quantification of the average thickness of the basal lamina of the blood vessels as described in (B), each point represents a separate blood vessel from 3 independent animals. The red lines represent the mean.

(D) Correlative light and electron microscopy of a 100μm thick vibrating microtome cross section of GFP-expressing Schwann cells (green) from a lectin (red) injected mouse sciatic nerve, Day 5 after transection. Panels on the left show a confocal image of GFP–expressing Schwann cells, either alone (top), overlayed on the correlated TEM image (middle) and TEM image alone (bottom). Outlined box highlights the GFP-expressing Schwann cell (SC) interacting with endothelial cells (EC), enlarged in the panels on the left, reconstructed in Figure 3G, and Movie S2. Note the points of direct contact between the Schwann cell and the blood vessel and sporadic/absent basal lamina of both cell types. Scale bars = 20 μm (white), 1 μm (black).

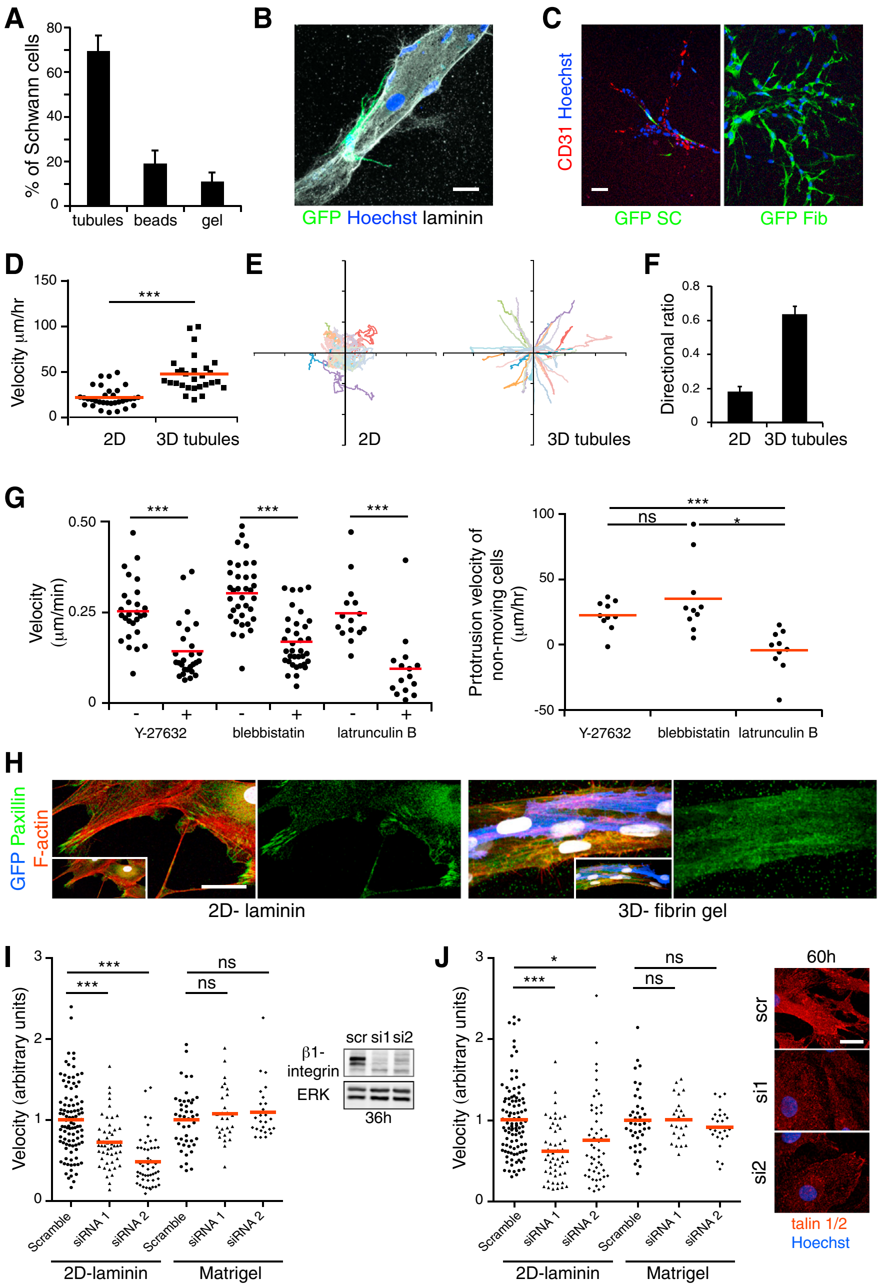

Figure S4. Primary Schwann Cells Migrate In Vitro along Tubules of Endothelial Cells, Related to Figure 4

(A) Quantification of the proportion of GFP-positive Schwann cells associated with the tubules of HUVECs, the beads or retained within the fibrin gel (n = 5 gels from separate experiments. 100 cells were counted per gel, graph shows mean value ± SEM).

(B) Representative confocal image of a GFP-positive Schwann cell (green) physically interacting with a laminin+ (white) tubule of endothelial cells, while migrating. Nuclei were counterstained with Hoechst (blue). Scale bar = 20 µm.

(C) Representative images of GFP-positive Schwann cells (left panel) or GFP-positive fibroblasts (right panel) co-cultured with endothelial tubules in fibrin gels. Scale bar = 50 µm. Schwann cells associate with the CD31+ endothelial cells whereas fibroblasts migrate within the matrix.

(D) Velocities of tracked single Schwann cells (dots) migrating on 2D laminin-coated surfaces or along tubules of HUVECs within fibrin gels. The red lines represent the mean.

(E) Single Schwann cell tracks migrating on 2D laminin-coated surfaces (left) or along tubules of HUVECs within fibrin gels (right). See also Movies S3 and S5.

(F) Directionality ratio of Schwann cells migrating on 2D laminin-coated surfaces or along tubules of HUVECs within fibrin gels. 30 cells were quantified in each condition from 3 separate experiments; graph shows mean value ± SEM.

(G) Left panel: Rear velocities of tracked single Schwann cells (dots) migrating along tubules of HUVECs upon inhibition with the inhibitors Y27632 (50 µM), blebbistatin (2 µM) or latrunculin B (0.2 µM). The red lines represent the mean. Right panel: Measurements of single Schwann cell protrusion velocities upon

(legend continued on next page)

inhibition with the inhibitors Y-27632, blebbistatin or latrunculin B. 10 cells were quantified from 2 separate experiments. The red lines represent the mean. One-way ANOVA test was used for statistical analysis. See also Movie S6. Note the movement of the rear of the cells is blocked by all three inhibitors whereas protrusions continue to form in the presence of Y-27632 and blebbistatin but not in the presence of latrunculin B.

(H) Representative confocal images of GFP-positive Schwann cells on a 2D-laminin surface or interacting with a tubule of HUVECs within a fibrin gel, immunostained for the focal adhesion complex marker paxillin (green) and labeled with phalloidin to visualize the cortical actin (red). Note that focal adhesion complexes are not detectable in Schwann cells migrating in 3D. Nuclei were counterstained with Hoechst (white). Scale bar = 25 μm.

(I) Left panel: Velocities of tracked single siRNA-treated Schwann cells (dots) migrating on 2D laminin-coated surfaces or along tubules of HUVECs in Matrigel. The red lines represent the mean. See also Movie S7. Right panel: Western blot analysis of total protein lysates from siRNA-treated Schwann cells showing the efficiency of beta1 integrin knockdown with two independent oligos compared to scrambled control, at 36 hr. Total ERK levels were used as a loading control.

(J) Left panel: Velocities of tracked single siRNA-treated Schwann cells (dots) migrating on 2D laminin-coated surfaces or along tubules of HUVECs in Matrigel. The red lines represent the mean. Right panel: Representative confocal images of talin 1 and 2 siRNA-treated Schwann cells immunostained for talin (red) showing the efficiency of talin 1 and 2 knockdown with two independent oligos at 60 hr. Scale bar = 50 μm.

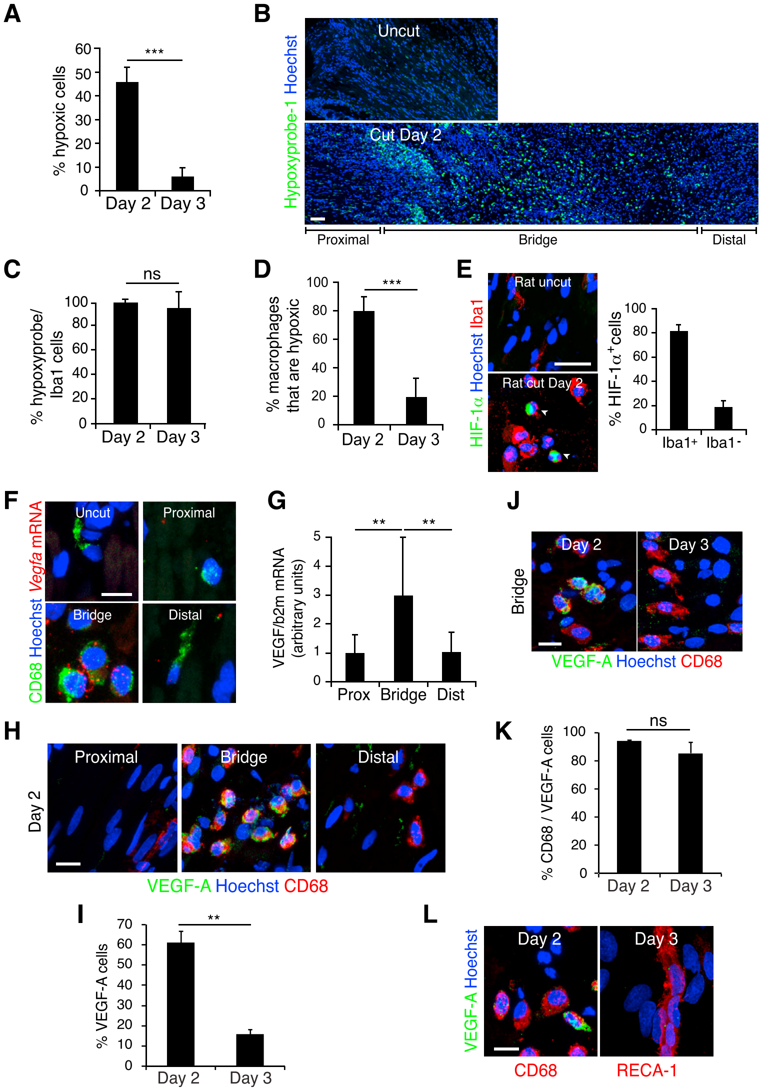

Figure S5. Hypoxia within the Bridge Leads to HIF-1α Stabilization and Expression of the Pro-angiogenic Target Gene, *Vegfa*, Related to Figure 5

(A) Quantification of Figure 5A to show the proportion of hypoxyprobe-1+ cells within the rat bridge (n = 4 animals per group; graph shows mean value ± SEM).

(B) Representative longitudinal sections of a rat sciatic nerve bridge and the contralateral uninjured nerve, Day 2 after transection and 30 min after injection of hypoxyprobe-1 (pimonidazole chloride), immunostained to detect hypoxyprobe-1 (green). Nuclei were counterstained with Hoechst (blue). Scale bar = 100 μm. To reconstruct the longitudinal section of the injured nerve (bottom), multiple images from the same sample were acquired using the same microscope settings.

(C) Quantification of Figure 5B to show the proportion of hypoxic cells that are macrophages at Day 2 and Day 3 (n = 4 animals per group; graph shows mean value ± SEM).

(D) Quantification of the proportion of macrophages (Iba1+) that are hypoxic (hypoxyprobe-1+), Day 2 and Day 3 after injury (n = 4 animals per group, graph shows mean value ± SEM). Note the significant decrease of hypoxic macrophages at Day 3 compared to Day 2.

(E) Representative images of a bridge region of a rat sciatic nerve, Day 2 after transection and the contralateral nerve (uncut), immunolabelled to detect macrophages (Iba1+, red) and HIF-1α expression (green). Scale bar = 20 μm. White arrowheads indicate HIF-1α+/Iba-1+ cells. Graph shows quantification of the proportion of HIF-1α+ cells that are macrophages (Iba1+) within the bridge (n = 3 animals, graph shows mean value ± SEM).

(F) Representative images of sections of rat sciatic nerve, Day 2 after transection and the contralateral uninjured rat sciatic nerve following in situ hybridization of rat *Vegfa* mRNA (red) and subsequent immunostaining for macrophages (CD68+, green). Scale bar = 10 μm.

(G) Quantitative RT-PCR analysis of *Vegfa* mRNA isolated from the bridge, the proximal and the distal stump of transected sciatic nerves, Day 2 after injury. Graph shows the *Vegfa* transcript levels relative to the levels in the proximal stump (n = 8 animals, graph shows mean value ± SEM).

(H–K) Representative images of cryosections of rat sciatic nerve, after transection, immunolabelled to detect VEGF-A (green) and macrophages (CD68+, red) to show proximal, bridge and distal regions at Day 2 (H) and the bridge region at Day 2 and Day 3 (J). The proportion of VEGFA+ cells (I) and VEGFA+ macrophages (CD68+) (K) are quantified at Day 2 and Day 3 in the bridge region (n = 3, graphs show mean value ± SEM).

(L) Representative images of a bridge region of a rat sciatic nerve, Day 2 or Day 3 after transection, immunostained to detect VEGF-A (green) and macrophages (CD68+, red) or blood vessels (RECA-1+, red) as indicated. Note that VEGF-A is undetectable in the blood vessels. Scale bars = 15 μm.

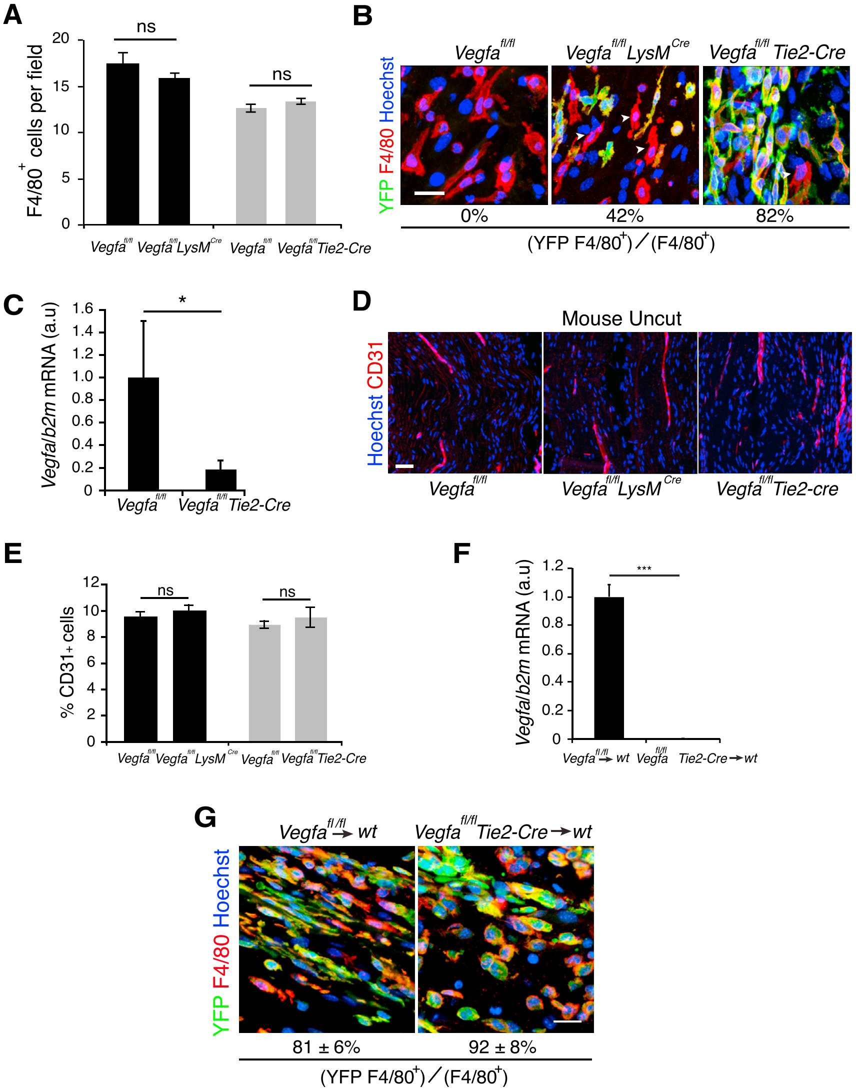

Figure S6. Inactivation of VEGF in Macrophages Inhibits Vascularization of the Bridge, Related to Figure 6

(A) Quantification of immunostained macrophages (F4/80+) within the bridge regions of *Vegfa*$^{fl/fl}$ *Lysm*^{Cre} mice, *Vegfa*$^{fl/fl}$ *Tie2-Cre* mice and their control littermates *Vegfa*$^{fl/fl}$, Day 5 after transection shows a similar recruitment of macrophages within control and mutant nerve bridges (n = 4 animals per group, graph shows mean value ± SEM).

(B) Representative images of bridges regions from *Vegfa*$^{fl/fl}$ (control), *Vegfa*$^{fl/fl}$ *Lysm*^{Cre} and *Vegfa*$^{fl/fl}$ *Tie2-Cre* mouse sciatic nerves immunostained to detect macrophages (F4/80+, red) and YFP (green), Day 5 after injury, to show the efficiency of recombination in macrophages. Scale bar = 20 μm. White arrowheads indicate examples of YFP-negative macrophages.

(C) Quantitative RT-PCR analysis of *Vegfa* mRNA levels in the bridge of *Vegfa*$^{fl/fl}$ *Tie2-Cre* mice relative to *Vegfa*$^{fl/fl}$ controls, Day 5 after injury (n = 4 for each group, graph shows the mean ± SEM).

(D) Representative images of longitudinal sections of uninjured sciatic nerves from *Vegfa*$^{fl/fl}$ (control), *Vegfa*$^{fl/fl}$ *Lysm*^{Cre} and *Vegfa*$^{fl/fl}$ *Tie2-Cre* mice, immunostained to detect endothelial cells (CD31+, red). Scale bar = 40 μm.

(E) Quantification of (D) showing the number of CD31+ endothelial cells in uninjured nerves of *Vegfa*$^{fl/fl}$ *Lysm*^{Cre} and *Vegfa*$^{fl/fl}$ *Tie2-Cre* animals as compared to their control littermates (n = 3 animals for each group, graph shows the mean ± SEM).

(F) Quantification of the levels of *Vegfa* mRNA in the bone marrow of wild-type mice transplanted with bone-marrow from *Vegfa*$^{fl/fl}$ (control) and *Vegfa*$^{fl/fl}$ *Tie2-Cre* mice. Bone marrow was extracted following the harvesting of the nerves, Day 5 following transection (n = 3); graph shows the mean ± SEM. The loss of *Vegfa* expression confirms both the efficiency of the bone marrow transplant (for additional information see Supplemental Experimental Procedures) and the efficiency of the recombination of the *Vegfa* locus in the cells derived from the *Vegfa*$^{fl/fl}$ *Tie2-Cre* mice.

(G) Representative images of bridge regions from mouse sciatic nerves Day 5 following transection from wild-type mice transplanted with bone-marrow from *Vegfa*$^{fl/fl}$ (control) and *Vegfa*$^{fl/fl}$ *Tie2-Cre* mice immunostained to detect macrophages (F4/80+, red) and YFP (green), to determine the percentage of macrophages in the bridge derived from the transplanted cells. These results confirm the efficiency of the bone marrow transplant (for additional information see Supplemental Experimental Procedures) and demonstrate that the vast majority of macrophages in the bridge are derived from the transplanted stem cells. Scale bar = 20 μm.

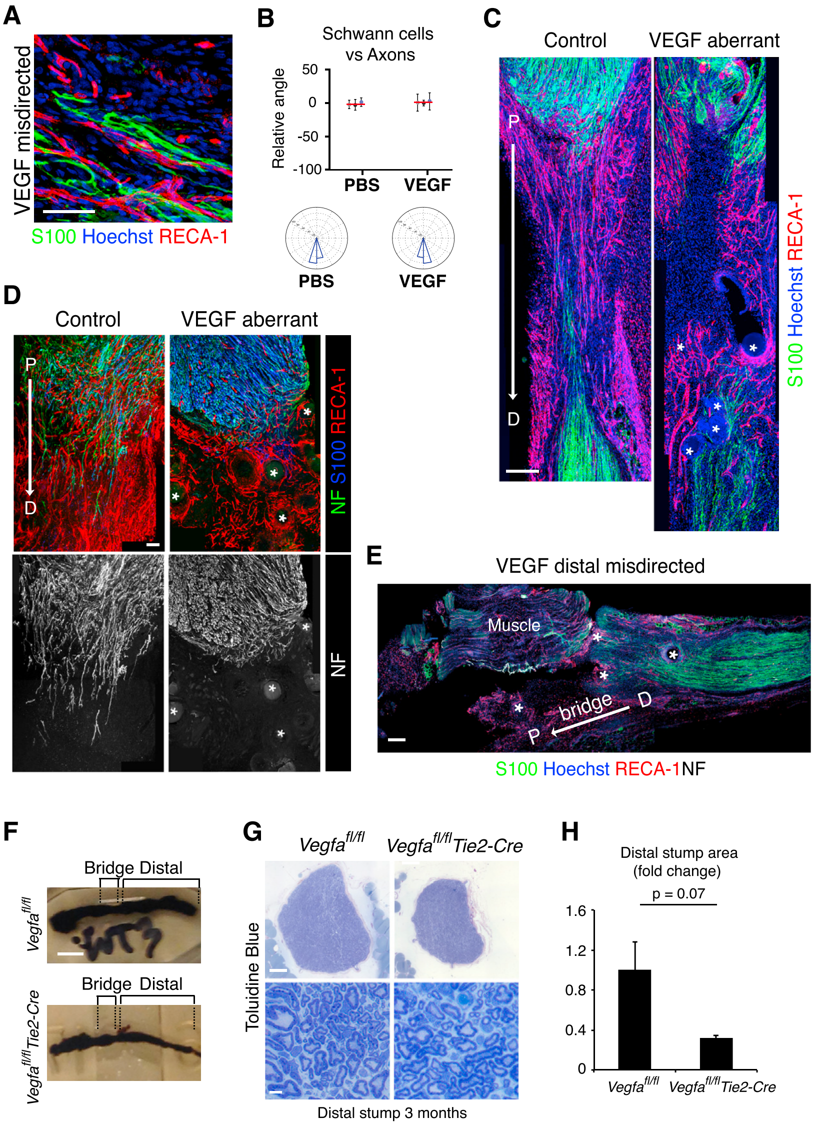

Figure S7. Disorganization of Blood Vessels Leads to Disrupted Schwann Cell Migration and Axonal Regrowth, Related to Figure 7

(A) A higher magnification of Figure 7B to show the Schwann cell cords (S100+, green), aligned to the blood vessels (RECA-1+, red). Scale bar = 50 μm.

(B) Quantification of Figure 7B to show the alignment between Schwann cells and regrowing axons. Graph shows the mean relative angle ± SD for each animal with the mean between animals shown by the red lines. Rose plots show the distribution of cells for all animals (n = 3 animals for each condition).

(C) Images of bridge regions of a control (PBS) and VEGF-treated rat sciatic nerve, Day 6 after injury, immunostained to detect Schwann cells (S100+, green) and endothelial cells (RECA-1+, red). Nuclei were counterstained with Hoechst (blue). Scale bar = 300 μm. The beads are indicated by white asterisks in the VEGF-treated animals. Note the center of the bridge is poorly vascularised in the VEGF-treated mice and the Schwann cell cords fail to enter the bridge.

(D) A further example of aberrant regeneration in a VEGF-treated sciatic nerve. Upper panels show images of a bridge region of control (PBS) and VEGF-treated rat sciatic nerves, Day 6 after injury, immunostained to detect Schwann cells (S100+, blue), endothelial cells (RECA-1+, red) and axons (NF+, green). Scale bar = 100 μm. Lower panels show the same images as in the upper panels, filtered to show only the axons (NF+, white). Note the axons in the VEGF-treated nerves are misdirected, toward the beads, into the adjoining muscle.

(E) Image of a disconnected nerve following treatment with VEGF-treated beads in which the beads redirect Schwann cell cords from the distal stump. Note the blood vessels (RECA-1+, red) and Schwann cells (S100+, green) are directed away from the bridge into the surrounding muscle. Scale bar = 200 μm. For reconstruction of longitudinal sections shown in (C), (D) and (E), multiple images from the same sample were acquired using the same microscope settings.

(F) Images of nerves stained with osmium tetroxide taken from *Vegfa$^{fl/fl}$* (control) and *Vegfa$^{fl/fl}$ Tie2-Cre* mice, 6 months following transection. Note the visibly smaller distal stump in the mutant mice. Scale bar = 2mm.

(G) Cross sections of a nerve from *Vegfa$^{fl/fl}$* (control) and *Vegfa$^{fl/fl}$ Tie2-Cre* mice 6 months following transection and stained with toluidine blue, at low magnification to show the entire nerve (top panels) and at higher magnification to show the indistinguishable structures of the control and mutant nerves (lower panels). Scale bar = 100 μm (top) and 5 μm (bottom).

(H) Graph to show the difference in area between the *Vegfa$^{fl/fl}$* (control) and *Vegfa$^{fl/fl}$ Tie2-Cre* nerves as in (G), n = 3; graph shows the mean ± SEM.

Cell

Supplemental Information

Macrophage-Induced Blood Vessels Guide Schwann Cell-Mediated Regeneration of Peripheral Nerves

Anne-Laure Cattin, Jemima J. Burden, Lucie Van Emmenis, Francesca E. Mackenzie, Julian J.A. Hoving, Noelia Garcia Calavia, Yanping Guo, Maeve McLaughlin, Laura H. Rosenberg, Victor Quereda, Denisa Jamecna, Ilaria Napoli, Simona Parrinello, Tariq Enver, Christiana Ruhrberg, and Alison C. Lloyd

Supplemental Experimental Procedures

In vivo analysis

Nerve injury

To induce the nerve transection injuries, left (rats) and right (mice) sciatic nerves were exposed under general anesthesia in aseptic conditions and transected with scissors at mid-thigh. Nerves were dissected at indicated time-points following transection for analysis. Cell proliferation was determined by measuring 5-ethynyl-2'-deoxyuridine (EdU) (Invitrogen) incorporation following a single intra-peritoneal injection of EdU (25mg/Kg body weight), 12 hours before sacrificing the animals.

In vivo inhibition of VEGFR

To inhibit VEGFR prior to vascularization, PLP-EGFP transgenic mice were orally administrated 3 doses of Cabozantinib (100mg/kg) (Bentzien et al., 2013; Yakes et al., 2011) or vehicle every 12 hours, Day 4 following sciatic nerve transection (1st dose in the morning) and sciatic nerves were collected the next day (Day 5, evening) to analyse blood vessel density in the bridge. To inhibit VEGFR post-vascularisation, PLP-EGFP mice were administrated the same doses, Day 5 following injury (1st dose in the morning) and sciatic nerves were collected the next day (Day 6, evening) to analyse Schwann cell migration and axonal regrowth. To test whether inhibition of bridge vascularisation has an effect on Schwann cell migration and axonal regrowth, PLP-EGFP mice were orally administrated 4 doses of Cabozantinib or vehicle every 12 hours, Day 4 following sciatic nerve transection (1st dose in the morning) and sciatic nerves were dissected on Day 7.

Rescue experiment

Day 4 after sciatic nerve transection of *Vegfa$^{fl/fl}$ Tie2-Cre* mutant mice, nerves were re-exposed under general anesthesia in aseptic conditions and each bridge was injected with 5μl of VEGF-A^{188} (ReliaTech GmbH) (4μg/ml) or PBS using a hand-held glass capillary coupled to a Hamilton syringe mounted to a microinjector (Sutter Instrument). One day later, sciatic nerves were collected for analysis.

VEGF-A treatment to misdirect blood vessels

Prior to surgery, agarose beads coated with heparin (Sigma) were pre-incubated with either PBS or human recombinant VEGF-A^{165} (1μg/ml) for 4 hours. The left sciatic nerves of rats were exposed under general anesthesia in aseptic conditions and transected at mid-thigh. VEGF or PBS beads were then placed to the side of the injury site within adjacent muscle. 6 days later, sciatic nerves were collected for analysis.

Bone marrow transplantation

3×10^6 total bone marrow cells from donor mice were injected into the tail vein of lethally irradiated (10 Gy) B6SJCD45.1 recipient mice. Donor cells were harvested from bone marrow from 3 *Vegfa$^{fl/fl}$ Tie2-Cre* mutant mice and 3 *Vegfa$^{fl/fl}$* littermates, which also constitutively expressed YFP in all cells. The donor cells were analysed by FACS for the expression of the CD45.2 locus and YFP reporter before transplantation into WT mice, which all expressed the CD45.1 locus to enable the analysis of the efficiency of the transplant. Bone marrow cells of each mouse were injected into the tail vein of 3 recipient mice. In order to check the engraftment efficiency, peripheral blood was collected from each recipient after 4 weeks of transplantation and analysed by FACS for CD45 locus and YFP expression. FACS analysis of peripheral blood and bone marrow was performed as previously described (Karlsson et al., 2013). Briefly, red blood cells were lysed using ACK buffer. Nucleated cells were stained with CD45.1 and CD45.2 antibodies conjugated with APC, PE, PEcy7 or APC-AF700. Hoechst 33358 was used to exclude dead cells. Stained cells were analysed on a Gallios (Beckman Coulter) flow cytometer.

Mice with higher than 90% engraftment of donor cells, as determined by expression of the CD45.2 locus, were used for sciatic nerve transection. The efficiency of the transplant was further confirmed by YFP-expression of bone marrow cells of transplanted animals and the high percentage of YFP+ macrophages in the bridge following injury.

In vitro analysis

Ex vivo bridge analysis

Sensitivity to hypoxia

Rat nerve bridges at Day 2 following injury were collected and digested with 1mg/ml dispase and 1mg/ml collagenase for 45 minutes at 37ºC, washed 3 times in RPMI (Gibco) containing 10% FCS and plated on PLL and fibronectin coated coverslips at 6 x10^4 cells/well in RPMI plus 10% FCS. After centrifugation at 300g for 5mins, cells were incubated overnight at 20% O_2, 5% CO_2. 100μM pimonidazole HCl (hypoxyprobe-1) was then added and the cells incubated at 20%, 1.5% or 0.1% O_2 for 4 hours before washing once with RPMI plus serum. Cells were then fixed in 4% paraformaldehyde (PFA) in PBS and immunostained for hypoxyprobe-1, Iba1 and the nuclei counterstained with Hoechst. For each experiment, each condition was in duplicate and 300 to 500 cells were counted per condition for each experiment.

Transwell migration assay

Cells from rat nerve bridges at Day 2 were purified as described above and seeded at 1x10^5 cells/well into the bottom of transwells overnight. The cells were then washed twice in

minimal media (RPMI containing BSA 100μg/ml, progesterone 60ng/ml, putrescine 16μg/ml, selenium 60ng/ml, thyroxine 50ng/ml, Triiodothyronine 50ng/ml, transferrin 100μg/ml, insulin 100ng/ml) and incubated at 1.5% O_2 for 24 hours. Minimal media was used as a control and 5ng/ml VEGF-A^{165} (Lonza) and 10% FCS were added as positive controls. Hanging cell culture inserts (8μm pore size, Millipore) were coated with fibronectin before adding Schwann cells or HUVECs ($7x10^4$ or $5x10^4$ cells respectively) in the presence or absence of 10μM Cabozantinib (Selleckchem) at 20% O_2. 4 hours later, cells on the upper surface were removed mechanically and cells that had migrated onto the lower surface were fixed in 4% PFA/PBS, the nuclei counterstained with Hoechst were then counted. 10 fields per condition were quantified, 5 from each duplicate and were normalised against migration in minimal media.

siRNA knockdown

siRNA knockdown was performed in primary rat Schwann cells using HiPerFect (Qiagen). siRNA target sequences used at the indicated concentrations were:
beta1 integrin siRNA 1 5'-ACAGCTGATTATCGATGCCTA-3' at 5nM
beta1 integrin siRNA 2 5'-CAAATTGTGGGTGGTGTACAA-3' at 5nM
Talin 1 siRNA 1 5'-CTGAGCGTCGTACAGAATCTA-3' at 3nM
Talin 1 siRNA 2 5'-CTGGTCGCTTGCAAGGTCAAA-3' at 3nM
Talin 2 siRNA 1 5'-ACCGGGCAAGCTTCTGACTAT-3' at 5nM
Talin 2 siRNA 2 5'-ACCCGAGGAATCAATAAGAAT-3' at 5nM
Following knockdown with beta1 integrin siRNA, cells were lysed in RIPA buffer (1% triton, 0.5% Na deoxycholate, 1mM EGTA, 50mM Tris pH 7.5) and standard Western Blotting for beta1 integrin was performed to validate the efficiency of the oligos at 36 hours. Following knockdown with talin 1 and talin 2 siRNA, cells were fixed and immunostained for talin to validate the efficiency of the oligos at 60 hours.

qPCR analysis

Nerve stump or nerve bridge samples were collected Day 2 or Day 5 following injury in rats and mice respectively and frozen in liquid nitrogen. Samples were crushed and homogenised on dry ice and then lysed in Trizol Reagent (Ambion). After RNA purification, RNA was reverse-transcribed using Super-Script II Reverse Transcriptase (Invitrogen). Quantitative PCR was then performed using the MESA Blue qPCR Kit (Eurogentec). Relative expression values for each gene of interest were obtained by normalizing to B2M (Napoli et al., 2012). Primers sequence used for qPCR were:

Rat Vegfa Fwd: 5'-gagttaaacgaacgtacttgcaga-3'; Rev: 5'-tctagttcccgaaaccctga-3'

Mouse Vegfa Fwd: 5'-agaaggagagcagaagtccca-3'; Rev: 5'-gtccaccagggtctcaatcg-3'

In situ hybridisation

12μm cryosections of contralateral or transected rat sciatic nerves at Day 2 were fixed for 20 minutes in 4% PFA/PBS and permeabilised with 0.3% Triton-X in PBS for 20 minutes at room temperature. Sections were then acetylated for 10 minutes and pre-hybridised in the hybridisation solution (Sigma) for 2 hours at room temperature. Hybridisation was performed using the scramble-ISH, the positive probe and the VEGF-A LNA-probe (5DigN/AGGGAAGTAGATCACAGAGGTT/3DigN) (Exiqon) at 14nM overnight at 55°C. After hybridisation, the sections were washed with 5X SSC for 5 minutes, 2X SSC for 1 minute, 0.2X SSC + 50% formamide for 30 minutes at 55°C and finally 0.2X SSC for 5 minutes at room temperature, incubated for 1 hour at room temperature in 1% blocking solution (Roche) prior to incubation with the CD68 antibody overnight at 4°C. Slides were then incubated with anti-DIG-AP, 488 conjugated anti-rat secondary antibody and Hoechst for 1 hour at room temperature. To detect the probe, sections were incubated with Fast Red (Roche) for 2 hours at room temperature. Images were detected using confocal microscopy.

Schwann cell migration assays

2D migration assay

$8x10^3$ siRNA treated GFP-labelled Schwann cells were seeded into laminin-coated wells and incubated overnight before starting time-lapse phase microscopy.

Fibrin gel bead assay

The fibrin gel bead assay was performed as described in the protocol of Nakatsu et al., 2003, with some modifications. Specifically, after coating the beads with HUVECs, the beads were resuspended in 2.5 mg/ml fibrinogen containing aprotinin at 0.15 U/ml. GFP-expressing rat Schwann cells were then added to the beads at a final concentration of $1.25x10^4$ cells/ml. 0.625 U/mL of thrombin was added to promote fibrin formation and then 400μl of the solution was added to a 10mm glass-bottom dish or a 24-well plate (MatTek Corporation). Time-lapse microscopy or immunofluorescence analysis was performed 10 days later. Rho kinase inhibitor (Y-27632, Abcam) was used at 50μM, myosin-II inhibitor blebbistatin (Abcam) at 2μM and latrunculin B (Sigma) at 0.2μM.

Matrigel assay

Briefly, 150μl matrigel/well (BD Bioscience) was coated on a 24 well plate on ice and polymerised at 37°C for 30 min. $2.5x10^4$ HUVECS were seeded onto the matrigel and incubated for 14 hours to allow tubules to form. $5x10^3$ Scrambled or siRNA treated GFP-

labelled Schwann cells were seeded onto the tubules and imaged using time-lapse microscopy.

Time-lapse microscopy and analysis

Live-imaging was performed using a Zeiss Axiovert 200M microscope equipped with a Hamamatsu Orca AG camera controlled by Volocity software (Improvision) and an environmental chamber which maintained the temperature at 37°C and provided a humidified stream of 5% or 10% CO_2 in air. Images were taken every 10 minutes for 12 to 24 hours for 2D and fibrin gel experiments. To compare single cell behaviour on 2D laminin-coated surfaces with 3D fibrin gels, cells were tracked using Volocity software for 8 to 12 hours and then speed and directional persistence were measured as previously described (Gorelik and Gautreau, 2014). In matrigel experiments, images were acquired every 10 minutes for 24 hours, cells were tracked the first 6 to 8 hours of acquisition and the velocity was measured using Volocity software. GFP-positive Schwann cells were seeded 36 hours and 60 hours following knockdown of beta1 integrin and talins respectively.

When fibrin gels were treated with inhibitors, images were taken every 7.5 minutes 6 hours prior to and 6 hours after addition of inhibitors. The rear of the migrating cells was tracked and the velocity was measured using Volocity software (27 cells were measured with Y-27632, 35 cells with blebbistatin and 15 cells with latrunculin B from 2 separate experiments). For protrusion measurements, the length of 2 to 5 protrusions were measured and averaged per cell at 0 and 3 hours in the presence of each inhibitor. The velocity of the protrusions was then calculated for each cell (10 cells from 2 separate experiments were measured in each condition).

Immunostaining

Sciatic nerves were dissected and fixed for 4 hours in 4% PFA/PBS, cryoprotected in 30% sucrose/PBS overnight at 4°C, incubated in 50% OCT/30% sucrose/PBS for 2 hours and finally embedded in OCT before being frozen in liquid nitrogen.

Longitudinal cryosections of contralateral or cut sciatic nerves at indicated times after transection were analysed in both rats and mice. Sections for immunostaining (12µm) or for quantification of blood vessel density, Schwann cell migration and axonal regrowth (40-60µm) were cut using a cryostat (Leica). Thin sections (12µm) were permeabilised in 0.3% triton/PBS for 30 minutes, washed and then blocked in 10% goat serum (Sigma)/PBS for 1 hour. Thick sections (40-60µm) were permeabilised and blocked in 0.3% triton 10% goat serum/PBS for 3 hours. Primary antibodies were diluted in 10% goat serum/PBS at the indicated concentration (see below) and incubated overnight at 4°C. After washing, the appropriate fluorescent secondary antibody (1/400, Alexa fluor®488, 594 or 647 from

Invitrogen) was used with Hoechst to counterstain the nuclei for 1 hour or 2.5 hours in thin (12μm) and thick (40-60μm) sections respectively. Samples were mounted in Fluoromount G (Southern Biotechnology). To determine the functionality of the blood vessels, fluorescein-conjugated *Griffonia* Simplicifolia lectin I (Vector Lab) was injected into the tail vein 10 minutes prior to harvesting of the animals. The hypoxyprobe-1 kit (hypoxyprobe^{TM}) was used to detect hypoxic cells according to the manufacturer's instructions. For HIF-1α immunostaining, nerves were directly embedded in OCT and snap frozen in liquid nitrogen. Cryosections were post-fixed in 4% PFA/PBS, incubated for 15 minutes in 0.1M Glycine/PBS and the above described protocol of immunostaining was performed with an additional tyramide signal amplification step (Invitrogen). For lipocallin-2 immunostaining, 6μm paraffin sections were cut using a microtome (Leica) and wide-field microscopy was used to acquire representative images. For the detection of EdU, nerve sections were stained using the Click-iT® EdU cell proliferation assay kit according to the manufacturer's instructions (Invitrogen).

For immunofluorescence of the fibrin gels, fixation with 4% PFA/PBS was performed for 5 hours at room temperature, followed by permeabilisation of the cells with 0.5% triton/PBS for 2 hours and blocking in 3% BSA/PBS for 5 hours under agitation at room temperature. Primary and secondary antibodies were incubated for 48 hours at 4°C. All fluorescent images were acquired using single photon confocal microscopy (Leica).

Primary antibodies

The following primary antibodies were used for immunofluorescence or for immunohistochemistry staining at the indicated dilutions: p75 (1/500, Millipore ab1554), RECA-1 (1/50, AbD Serotec MCA970), CD31 (1/300, BD Pharmingen 553370), S100 (1/100, Dako Z0311), IBA1 (1/500, Wako 019-19741), mouse-F4/80 (1/500 AbD Serotec MCA497), rat-CD68 (1/100, AbD Serotec MCA5709), prolyl-hydroxylase beta (1/1000, Acris), GFP (1/500, Abcam ab13970), neurofilament 200 kD (1/1000, Abcam ab4680), HIF-1α (1/100, Novus Biologicals NB 100-1231), alkaline-phosphatase-conjugated dioxygenin or AP-DIG (1/1000, Roche), VEGF (1/200 Abcam ab46154), elastin (1/500, Abcam ab21610), fibronectin (1/1000, sigma clone FN-3E2), laminin (1/500, Abcam 11575), collagen I (1/500, Abcam ab292), collagen IV (1/500, Abcam ab19808), paxillin (1/500, Abcam ab32084), talin (1/500, sigma T3287) and beta1 integrin for western (1/500, Abcam ab52971).

Toluidine Blue staining

Sciatic nerves were fixed with 2% glutaraldehyde in 0.2 M phosphate buffer overnight at 4°C, post-fixed in osmium tetroxide for 2 hours at 4°C and then in 2% uranyl acetate for 45 minutes at 4°C. Nerves were then dehydrated in an ethanol series before embedding in

epoxy resin. Semithin sections were cut with a glass knife at 0.1μm, dried carefully and stained with 0.5% toluidine blue in 2% borax at 75°C for 30 seconds. After dehydration, sections were mounted with DPX (Sigma) and representative images were acquired using wide-field microscopy.

Confocal microscopy and image analysis

All fluorescent images were acquired using inverted SPE confocal microscopes (Leica).

For the reconstruction of complete 40-60μm longitudinal sections of sciatic nerves, the section corresponding of the middle of the nerve was selected and images were acquired from the proximal to the distal stump. For each experiment, the same volume and number of z-stacks was taken and the same settings of acquisition were used. For each nerve, a projection of the z-stacks was made using Fiji software (http://fiji.sc/Fiji). The projections were then merged using Adobe Photoshop software.

For blood vessel, Schwann cell and axon area measurements, the reconstructed images were converted to 8-bit grey scale TIFF using Fiji software, the area corresponding to the nerve bridge was then selected, thresholded and made binary. The Create Selection function was used to automatically outline the thresholded area and then the immunostained area was quantified using the measurement function.

For alignment measurements (Figures 7C-E) relative to the nerve proximal to distal axis, NeuronJ was used to draw the cords of Schwann cells or the regrowing axons and Fiji was used to draw the axis of the blood vessels on reconstructed sciatic nerves. The angle of incidence was measured between the proximal to distal nerve axis and blood vessels (300 blood vessels measured per condition from 3 separate animals), Schwann cells (200 cords measured per condition from 3 separate animals) and axons (200 regrowing axons measured per condition from 3 separate animals). For alignment measurements between either Schwann cells and blood vessels or Schwann cells and axons (Figures 7F and S7B), confocal images acquired from the leading edge of migration in the bridge were used to draw the axis of the blood vessels, the cords of Schwann cells and the axons. The angle of incidence was measured between the cell types (100 angles were measured per condition from 3 separate animals of the alignment between Schwann cells and blood vessels and 150 angles were measured from 3 separate animals to determine the alignment of the Schwann cells and axons). In the graphs, each point represents the mean angle for each of the 3 animals +/- SD and the red line represents the mean between the animals for each condition. Rose plots were constructed using Matlab.

Confocal 3-D reconstructions

Figure 3E: Initial reconstruction was carried out in Fiji to identify the blood vessel of interest. The stack was processed to remove background and noise, then cropped and imported into BioVis3D (http://www.biovis3d.com/). Structures were created for each blood vessel interacting with the selected Schwann cell, reconstructed using a quick auto-contour selection tool, then manually adjusted to account for any discrepancies, before creating a 3D surface rendering of a z confocal stack. The nuclei of the selected Schwann cell and the interacting blood vessels were added using manual contour tracing. Snaps were taken at 1024x1024 300dpi at selected angles. Figure 3H: After importation of the LIF file into Imaris software (http://www.bitplane.com/go/products/imaris), the channels corresponding to the blood vessels (blue), the Schwann cells (green) and the axons (red) underwent noise removal. Three surfaces were created, one for each type of structure. Default settings were used in surface wizard, minor adjustments to the intensity threshold were made for Schwann cells and blood vessels to help pick up the dimmer signal. The results were then manually filtered to remove noise in the Schwann cell and axon channels. The images shown in Figure 3H were made using the movie setting on Imaris to calculate the angle and then captured in a snapshot at 1024 x1024 300dpi.

Correlative light and electron microscopy (CLEM)

CLEM – 3D fibrin gel culture: Briefly, GFP-positive Schwann cells in contact with endothelial tubules within the fibrin gel were identified using a fluorescence widefield microscope and imaged together with their location in relation to a photo-etched grid (MatTek Corporation), by fluorescence and brightfield imaging. Cells were then fixed in 4% PFA/PBS overnight and the region of interest was relocated and a razor blade was used to remove all other fibrin gel and cells from the dish to aid infiltration of fixatives and reagents. The samples were then further fixed, dehydrated and embedded in Epon (Stinchcombe et al., 1995). Light microscopy images (xy) were then used to re-locate the GFP-positive Schwann cell in contact with the endothelial tubule in the resin block. For TEM analysis, 70nm sections of the endothelial tubule cut in cross-section (xz) were stained with lead citrate and imaged in a Tecnai 20 (FEI) using a Morada camera and iTEM software (Olympus SIS).

CLEM – 3View: For serial block face imaging, the region of interest was excised, mounted onto a specimen pin and coated with gold palladium before being imaged and sectioned using 3View (Gatan) coupled to a Sigma FEG-SEM (Zeiss). CLEM – ex-vivo: Sciatic nerves from PLP-EGFP mice were collected 10 minutes after tail vein injection of Rhodamine conjugated *Griffonia* Simplicifolia lectin I (Vector Lab), fixed overnight in 4%PFA/PBS at 4°C and embedded in 2.8% low melting point agarose dissolved in L15 media. 100µm thick cross-sections were then cut in cold PBS using a vibrating microtome and serial sections were collected throughout the bridge region of the nerve. Sections were screened using a widefield

fluorescence microscope to identify sections of the nerve bridge where GFP-positive migrating Schwann cells were exiting the stumps. These sections were then imaged at higher magnification using a confocal microscope and full z-stacks were acquired. Sections were then further fixed, dehydrated, and embedded in Epon. Ultrathin sections (70nm) were taken of the whole nerve, and GFP-positive Schwann cells and blood vessels were mapped using both the fluorescence and electron microscopy datasets. More specific regions of interest were identified, and the blocks were further trimmed to allow for collection of serial sections. Serial sections were aligned, cells of interest segmented, rendered and reconstructed in 3D using Amira (FEI).

Supplemental References

Bentzien, F., Zuzow, M., Heald, N., Gibson, A., Shi, Y., Goon, L., Yu, P., Engst, S., Zhang, W., Huang, D.*, et al.* (2013). In vitro and in vivo activity of cabozantinib (XL184), an inhibitor of RET, MET, and VEGFR2, in a model of medullary thyroid cancer. Thyroid *23*, 1569-1577.

Gorelik, R., and Gautreau, A. (2014). Quantitative and unbiased analysis of directional persistence in cell migration. Nature protocols *9*, 1931-1943.

Karlsson, G., Rorby, E., Pina, C., Soneji, S., Reckzeh, K., Miharada, K., Karlsson, C., Guo, Y., Fugazza, C., Gupta, R.*, et al.* (2013). The tetraspanin CD9 affords high-purity capture of all murine hematopoietic stem cells. Cell Rep *4*, 642-648.

Napoli, I., Noon, L.A., Ribeiro, S., Kerai, A.P., Parrinello, S., Rosenberg, L.H., Collins, M.J., Harrisingh, M.C., White, I.J., Woodhoo, A.*, et al.* (2012). A central role for the ERK-signaling pathway in controlling Schwann cell plasticity and peripheral nerve regeneration in vivo. Neuron *73*, 729-742.

Stinchcombe, J.C., Nomoto, H., Cutler, D.F., and Hopkins, C.R. (1995). Anterograde and retrograde traffic between the rough endoplasmic reticulum and the Golgi complex. J Cell Biol *131*, 1387-1401.

Yakes, F.M., Chen, J., Tan, J., Yamaguchi, K., Shi, Y., Yu, P., Qian, F., Chu, F., Bentzien, F., Cancilla, B.*, et al.* (2011). Cabozantinib (XL184), a novel MET and VEGFR2 inhibitor, simultaneously suppresses metastasis, angiogenesis, and tumor growth. Mol Cancer Ther *10*, 2298-2308.
